# 1D and 2D NMR datasets, resonance assignments and coupling constant analysis of red beet fiber and pectin

**DOI:** 10.1016/j.dib.2022.108845

**Published:** 2022-12-20

**Authors:** Gary D. Strahan, Arland T. Hotchkiss, Senghane Dieng, Julie Hirsch

**Affiliations:** a Agricultural Research Service, U.S. Department of Agriculture, Dairy & Functional Foods Research Unit, 600 E. Mermaid Lane, Wyndmoor, PA 19038 United States of America; b Ingredion, Inc., 10 Finderne Avenue, Bridgewater, NJ 08807 United States of America

**Keywords:** NMR, Red beet, Fiber, Polysaccharides, Pectin

## Abstract

The datasets presented in this article represent detailed NMR spectral analyses on red beet fiber, including the pomace, water-soluble and water-insoluble fractions, as well as the acid-extracted pectin. The samples were solvated in deuterium oxide and investigated by 1D-^1^H, 1D-^13^C NMR, and multiple 2D-NMR experiments, including gCOSY, zTOCSY, HSQC, HMBC, HSQCTOCSY, and H2BC. The NMR chemical shifts, coupling constants and spin-systems were identified for the major carbohydrate residues in each sample. This article provides additional data related to the research article “Structural characterization of red beet fiber and pectin” published in Food Hydrocolloids [1].

**Specifications Table**

**Table:** 

Subject	Chemistry
Specific subject area	Structural analysis
Type of data	NMR spectra, assignments, and FID files
How the data were acquired	14 Tesla Agilent DD2 NMR spectrometer (Santa Clara, CA) using a 5 mm OneNMR probe with z-axis pulsed field gradients. All NMR spectra were processed using [2] and visualized using UCSF Sparky.
Data format	Analysed and raw
Description of data collection	Four fractions derived from red beet, including its pomace (PF), water-soluble (WSF), water-insoluble (WIF), and pectin, were dissolved in D_2_O with d_4_-trimethylsilylpropanoic acid (TMSP) added as an internal reference standard and sodium azide (NaN_3_) added as a preservative. Their NMR spectra were acquired at 40°C and 75°C.
Data source location	• Eastern Regional Research Center/USDA-Agricultural Research Service • Wyndmoor, PA • United States of America
Data accessibility	Analysed data are available within this article. Raw data are accessible at: https://fdc.nal.usda.gov/portal-data/external/experimentalSupplements/Strahan-Hotchkiss-RedBeet-WSF.zip https://fdc.nal.usda.gov/portal-data/external/experimentalSupplements/Strahan-Hotchkiss-RedBeet-Pectin.zip https://fdc.nal.usda.gov/portal-data/external/experimentalSupplements/Strahan-Hotchkiss-RedBeet-WIF.zip https://fdc.nal.usda.gov/portal-data/external/experimentalSupplements/Strahan-Hotchkiss-RedBeet-PF.zip
Related research article	Data in this article is associated with the paper: Arland T. Hotchkiss, Jr., Hoa K. Chau, Gary D. Strahan, Alberto Nuñez, Stefanie Simon, Andre K. White, Senghane Dieng, Eugene R. Heuberger, Madhav P. Yadav, and Julie Hirsch. (2022). Structural characterization of red beet fiber and pectin. Food Hydrocolloids. 10.1016/j.foodhyd.2022.107549

## Value of the Data

• The following data provides information on the NMR characterization of red beet fiber samples with different solubilities.
• The NMR observables, such as chemical shifts, coupling constants and correlations, can be useful for the assignment and interpretation of NMR spectra of similar fiber polysaccharides in different solvent conditions.
• This data may provide insights and understanding of the molecular fine structures of other polysaccharides and evaluate their functional properties in food systems.

## 1 Data Description

### 1.1 Data

The data provided here include the 1D and 2D NMR spectra of four red beet fiber fractions, the pomace (PF), water-soluble (WSF), water-insoluble (WIF), and microwave-extracted pectin (10/80 min/˚C). The anomeric ^1^H-^1^H coupling constants and chemical shift resonances of the observed carbohydrate residues are reported. Additional analyses can be found in the associated paper: “Structural characterization of red beet fiber and pectin” [1].

**Fig. 1:**
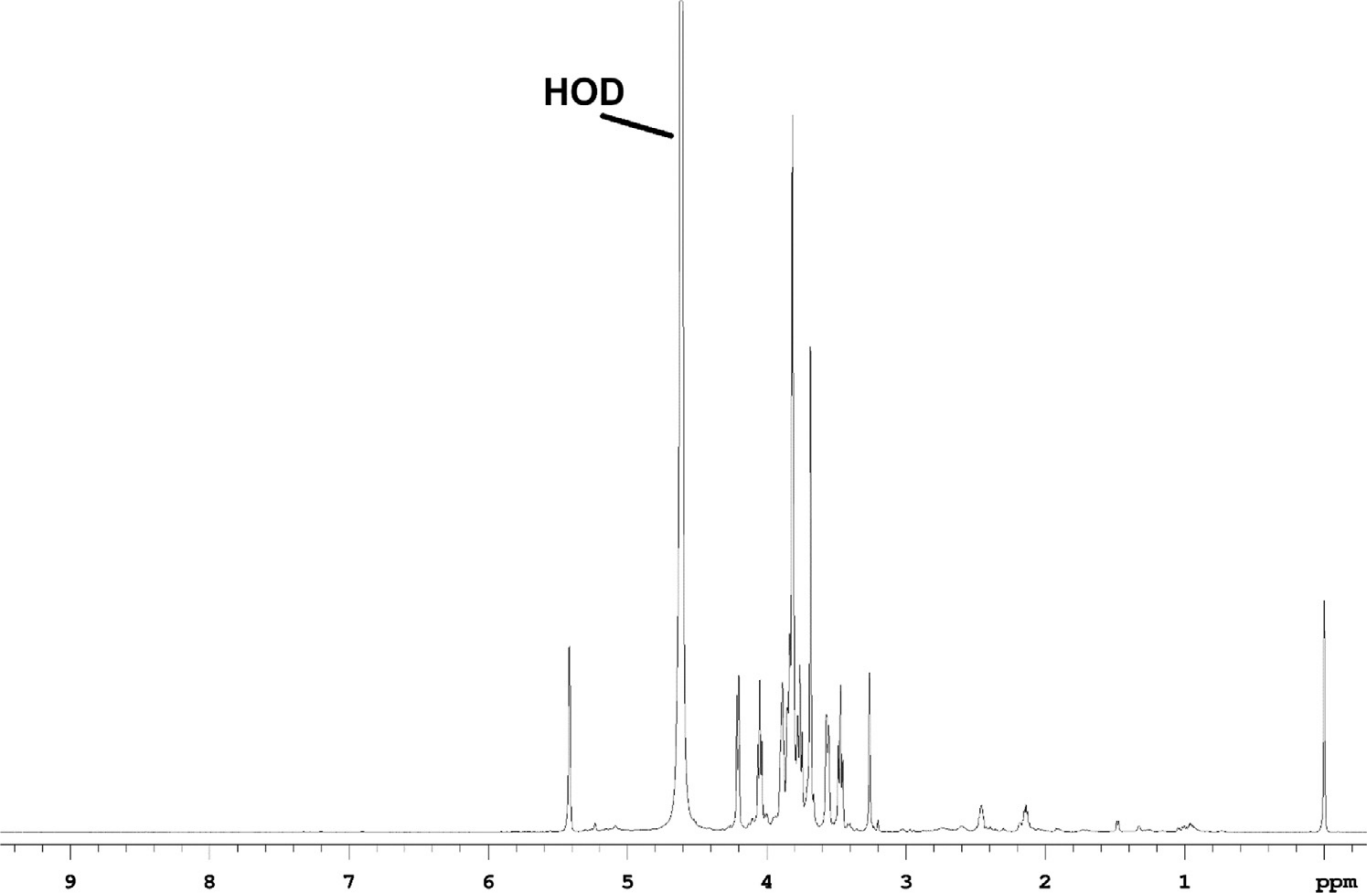
The 1D-^1^H spectrum of red beet water-soluble fraction.

**Fig. 2:**
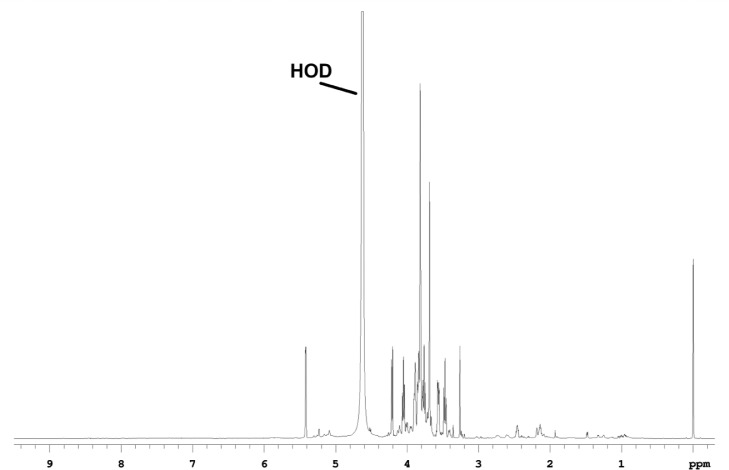
The ^1^H-1D NMR spectrum of red beet pomace fraction.

The dominant anomeric resonances in the 1D-^1^H NMR spectrum of the WSF (Fig. 1), as well as the PF (Fig. 2) are at 5.42 ppm, with smaller intensity resonances at 5.23 and 5.09 ppm, followed by increasingly weaker resonances at 5.16, 5.26, 4.56, 5.31 and 5.13 ppm. The resonance at 5.42 ppm is an apparent doublet with a *J*_H1H2_ coupling constant of 2.8∼3.42 Hz, which is consistent with H2 and H1 being in an axial-equatorial, or α conformation. These peaks are assigned as α-Glc(1) and α-Glc(2). Similarly, the weak resonance at 5.26 ppm is a doublet with J_H1H_=3.15 Hz, which is also α conformation, and is assigned as α-Glc(3). The weak doublet centered at 4.56 ppm has a J_H1H2_ = 7.51 Hz, which indicates an axial-axial orientation, or β conformation, and is assigned as β-Glc(2).

**Fig. 3:**
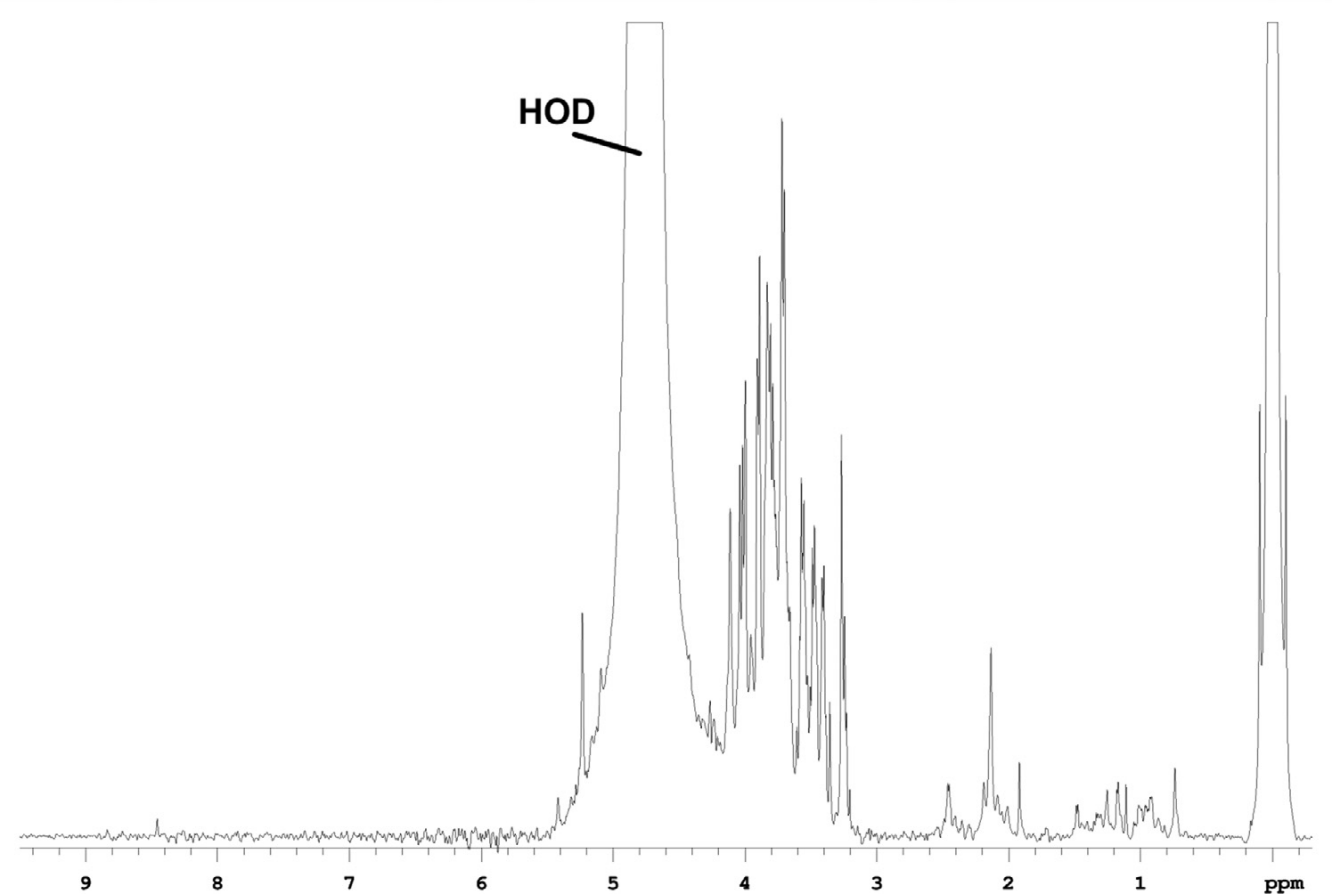
The 1D-^1^H NMR spectrum of red beet water-insoluble fraction.

In the 1D-^1^H NMR spectrum of red beet WIS (Fig. 3), the dominant resonance is at 4.65 ppm (doublet, *J*_H1H2_ = 8.1 Hz, β conformation) and is assigned as β-Glc(1); this resonance is obscured by the water HOD peak in the water-soluble and pomace fractions. The next most intense peak in the water-insoluble fraction is at 5.24 ppm (doublet, *J*_H1H2_ = 3.8 Hz, α conformation) which is assigned as α-Glc(3). Weaker peaks are found at 5.10 ppm (broad, *J*_H1H2_ = small, α conformation), which is assigned as α-Ara, and at 4.52 ppm (doublet, *J*_H1H2_ = 7.5 Hz, β conformation), which is assigned as β-Glc(2). The weakest anomeric peak observed is at 5.42 ppm (doublet, *J*_H1H2_ = 4.4 Hz, α conformation), arising from α-Glc(2).

**Fig. 4:**
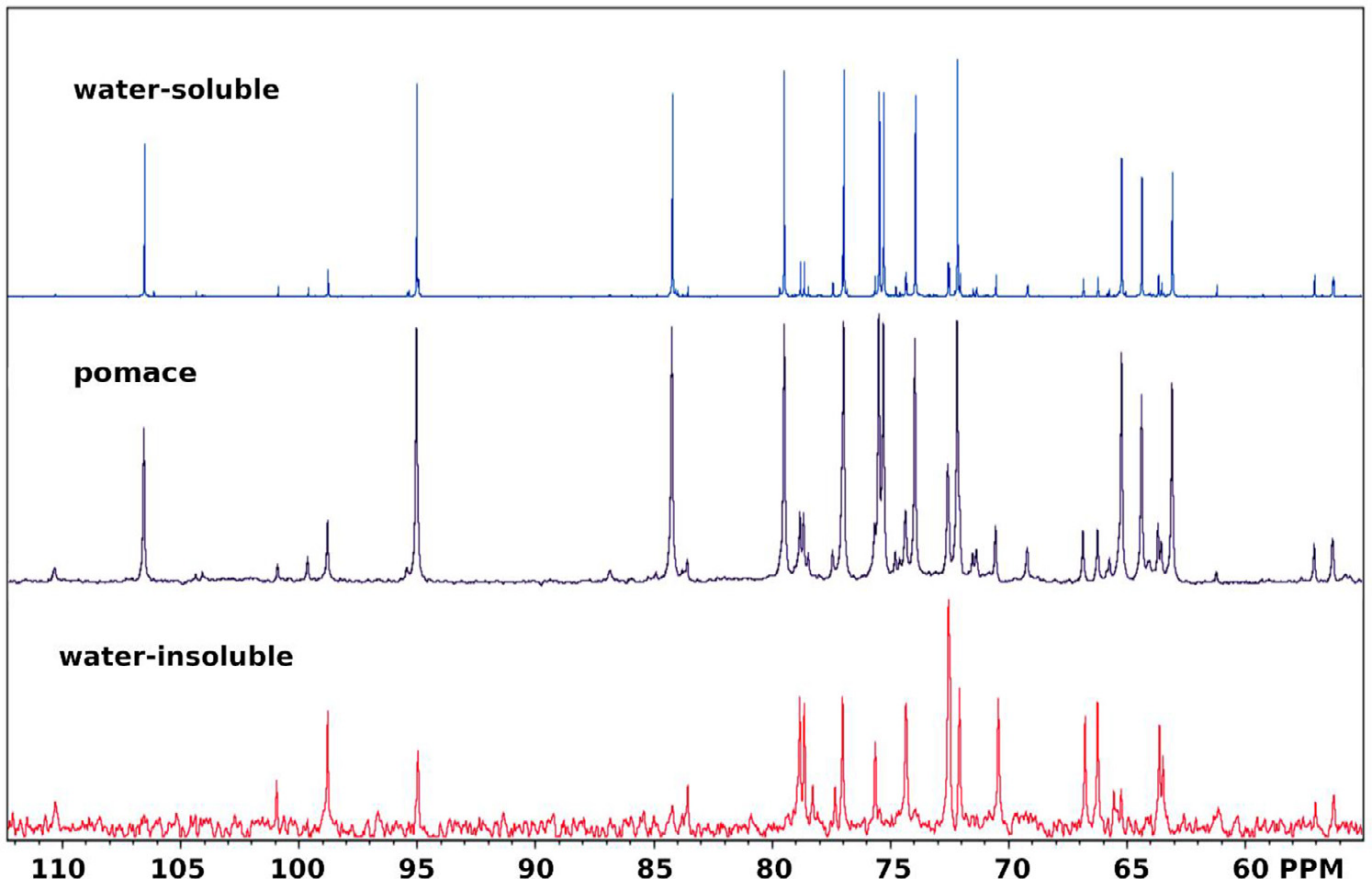
The sugar resonance region of the 1D-^13^C NMR spectra measured for water-soluble (top), pomace (middle) and water-insoluble (bottom) fractions.

All of the fractions contain many non-sugar resonances (0.5-3 ppm) of their 1D-^1^H spectra (Figs. 1-3), that are primarily aliphatic, and comprise about 10% of the proton spectral intensity in WSF and PF, and about 13% of the proton spectral intensity in the WIF. Comparing the sugar resonance regions of the 1D-^13^C NMR spectra (Fig. 4) for WSF, PF and WIF, it is seen that WSF and PF have very similar spectra, both in terms of resonances and intensities. The WIF 1D-^13^C spectrum, however, the resonances associated with Ara (∼110 ppm) and Glc (∼99 ppm) are not observed, as well as decreased sucrose resonances (Glc at ∼95 ppm and Fru at ∼84 ppm). All of these resonances can be weakly detected in the 2D spectra, however, indicating that while their concentration is diminished, they are still present.

**Fig. 5:**
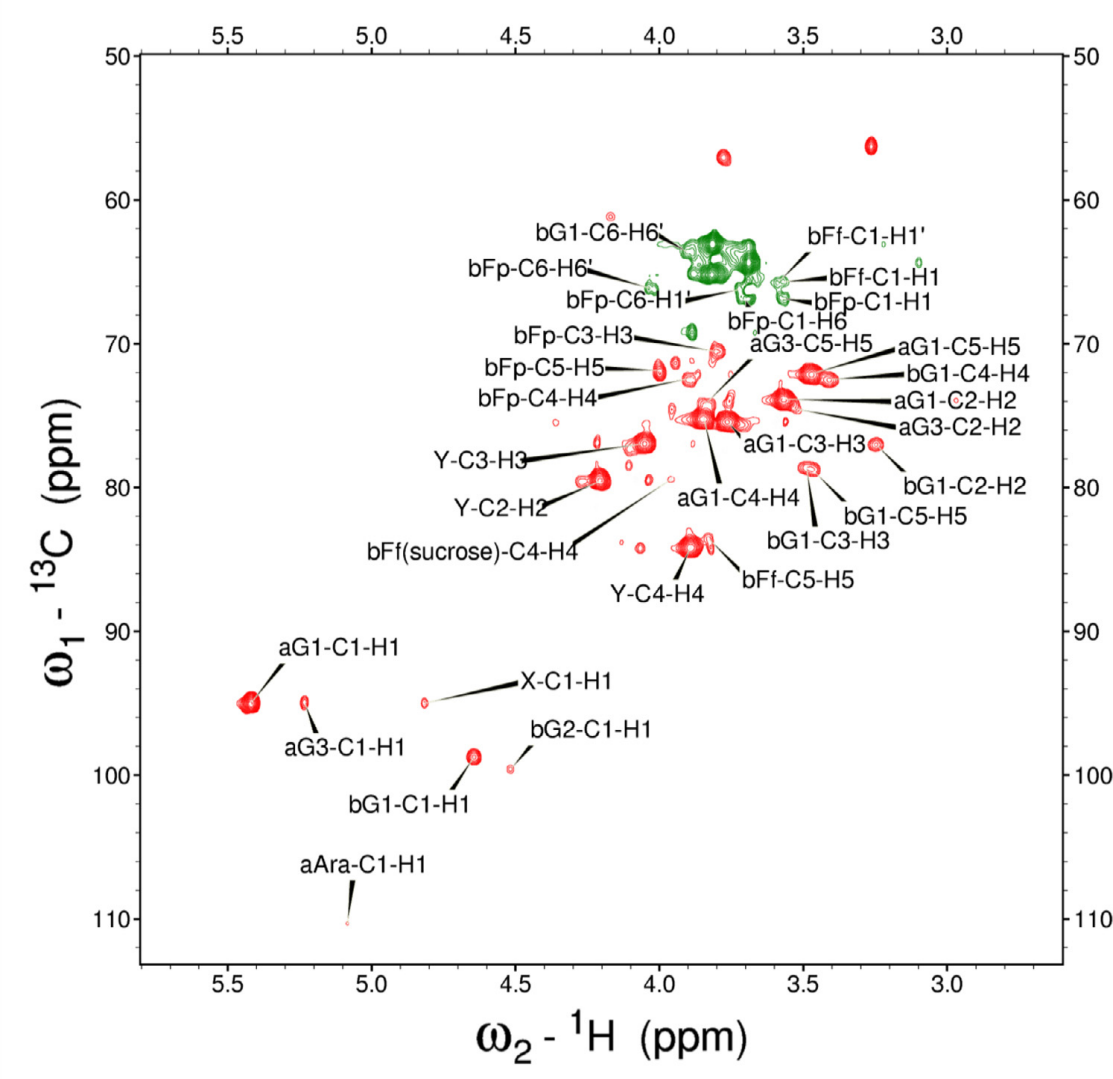
The multiplicity-edited 2D HSQC NMR spectrum of the sugar region in the red-beet water-soluble fraction with assignments. Red colored peaks indicate carbons with odd numbers of attached hydrogens, while green indicates a CH_2_ group.

**Fig. 6:**
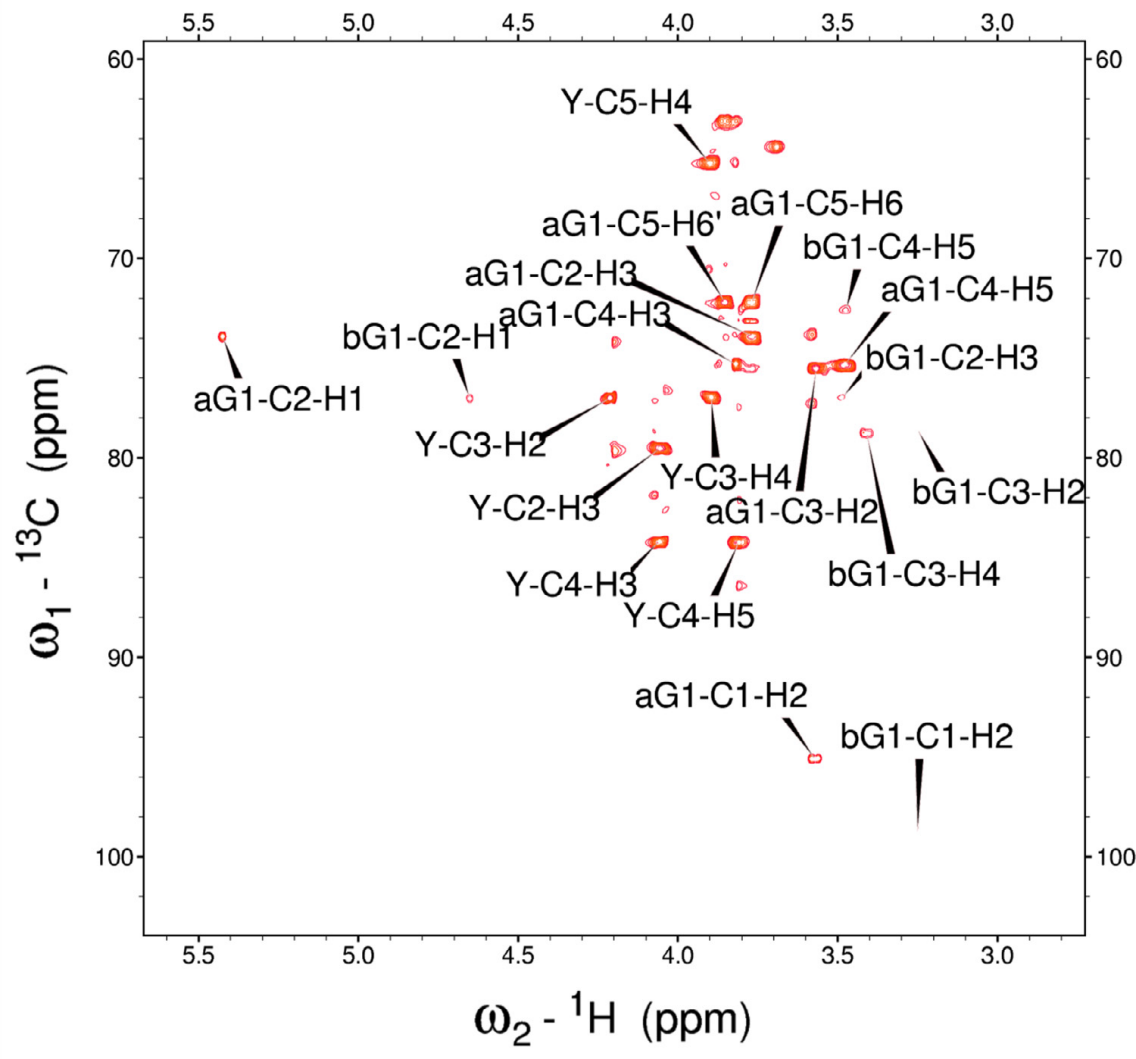
The H2BC NMR spectrum of the red beet water-soluble fraction with assignments.

**Fig. 7:**
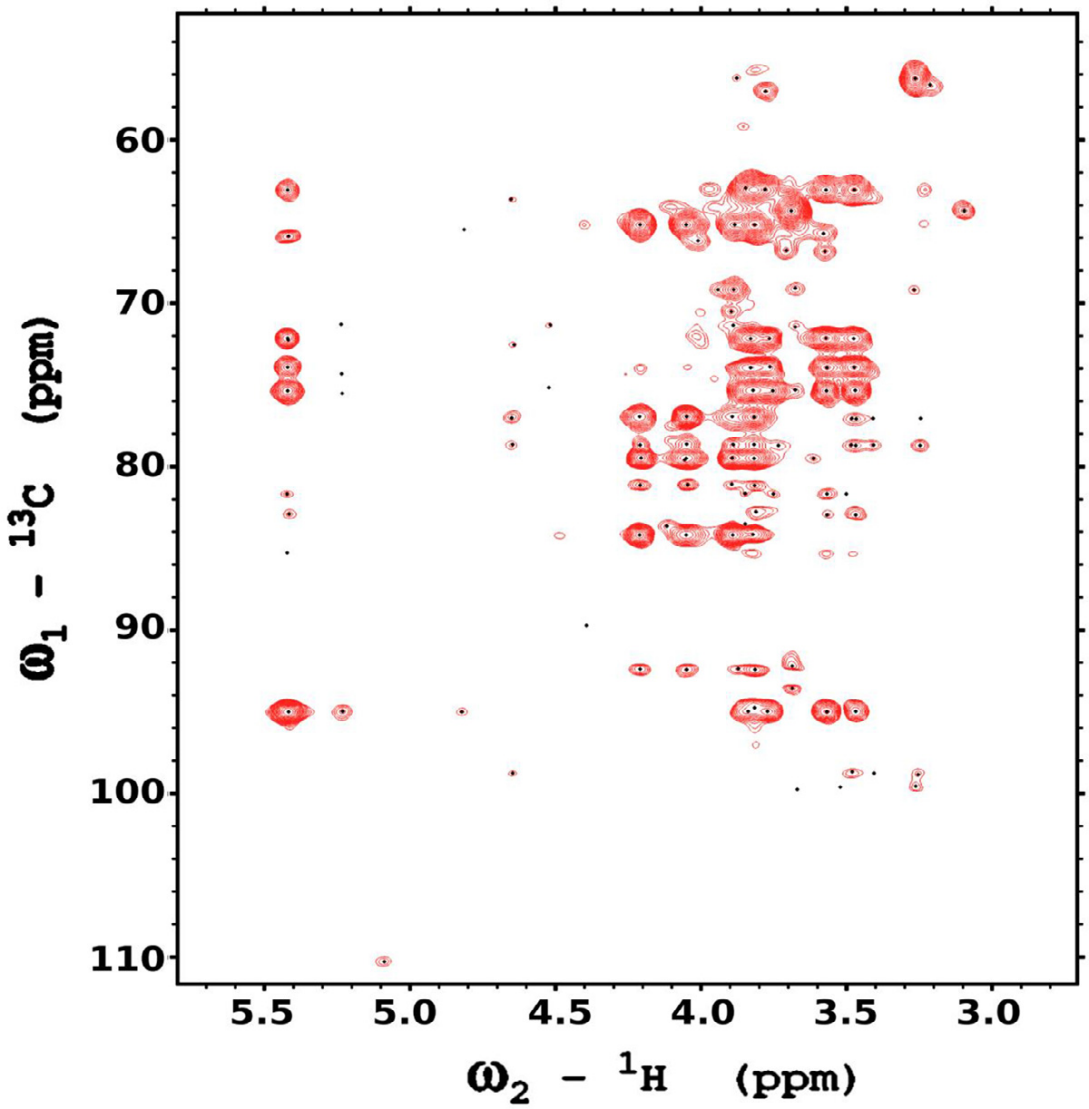
The 120 ms HSQCTOCSY NMR spectrum of the red beet water-soluble fraction.

**Fig. 8:**
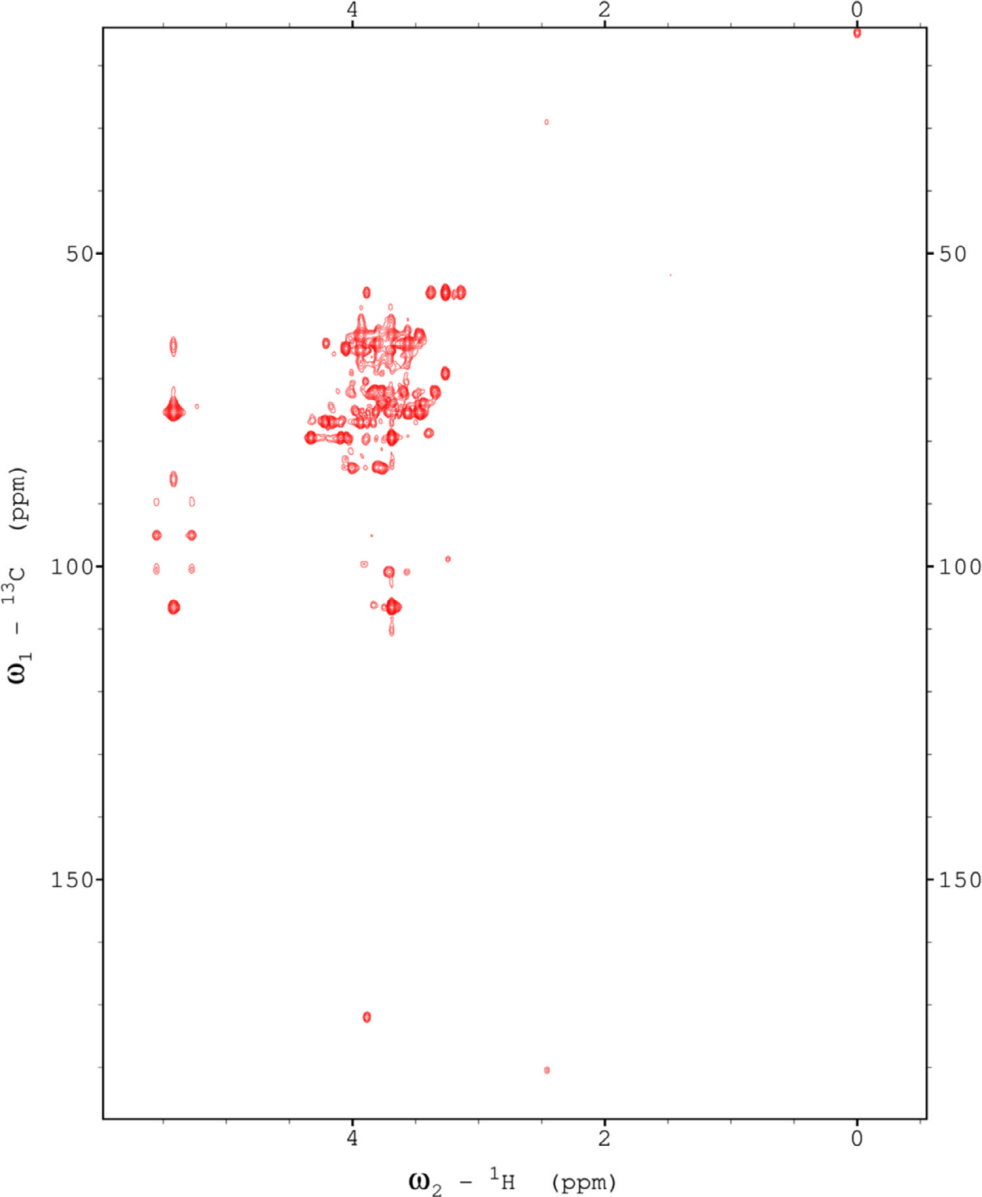
The 10 Hz HMBC NMR spectrum of the red beet water-soluble fraction.

**Fig. 9:**
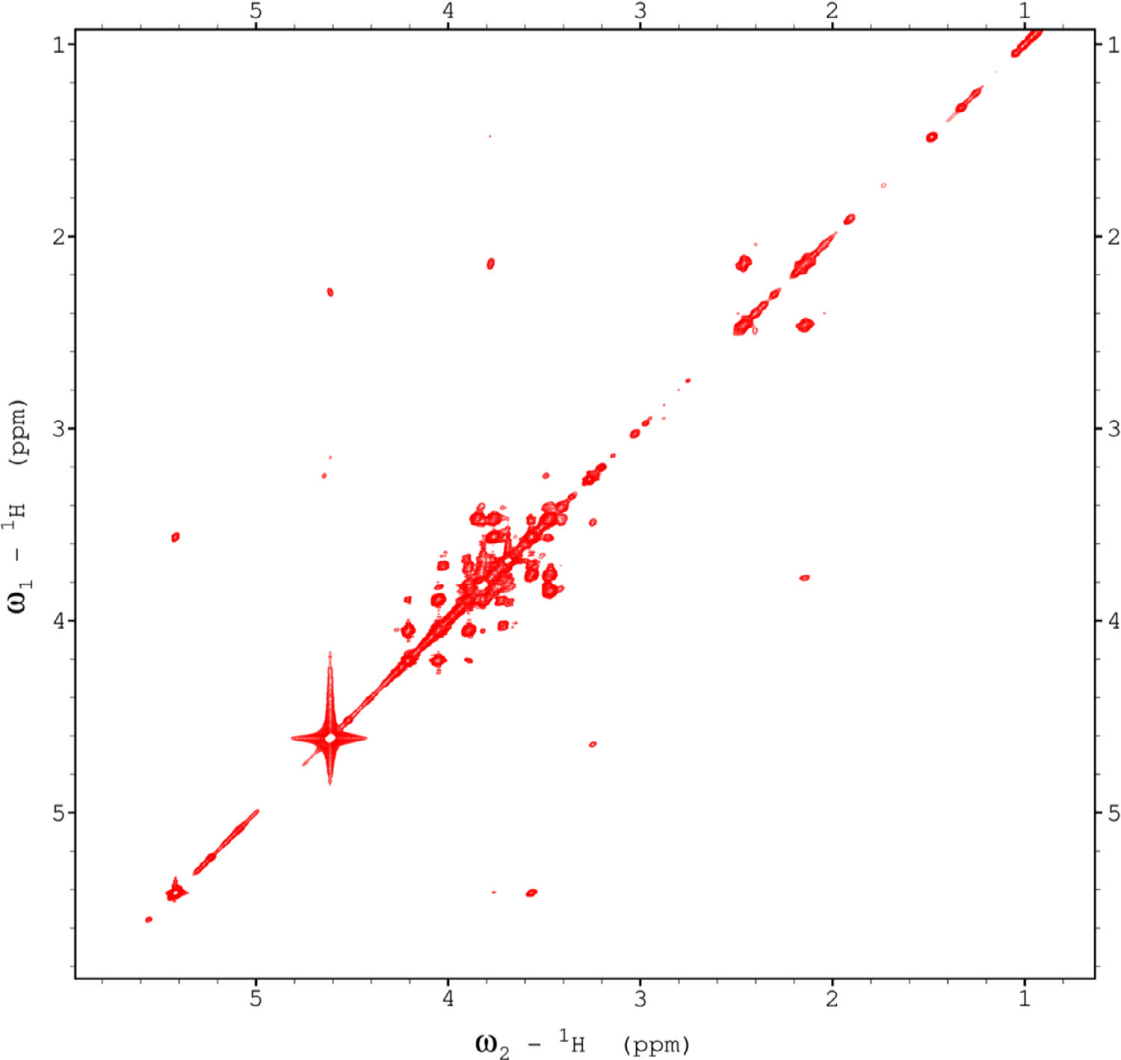
The 22ms zTOCSY NMR spectrum of the red beet water-soluble fraction.

**Fig. 10:**
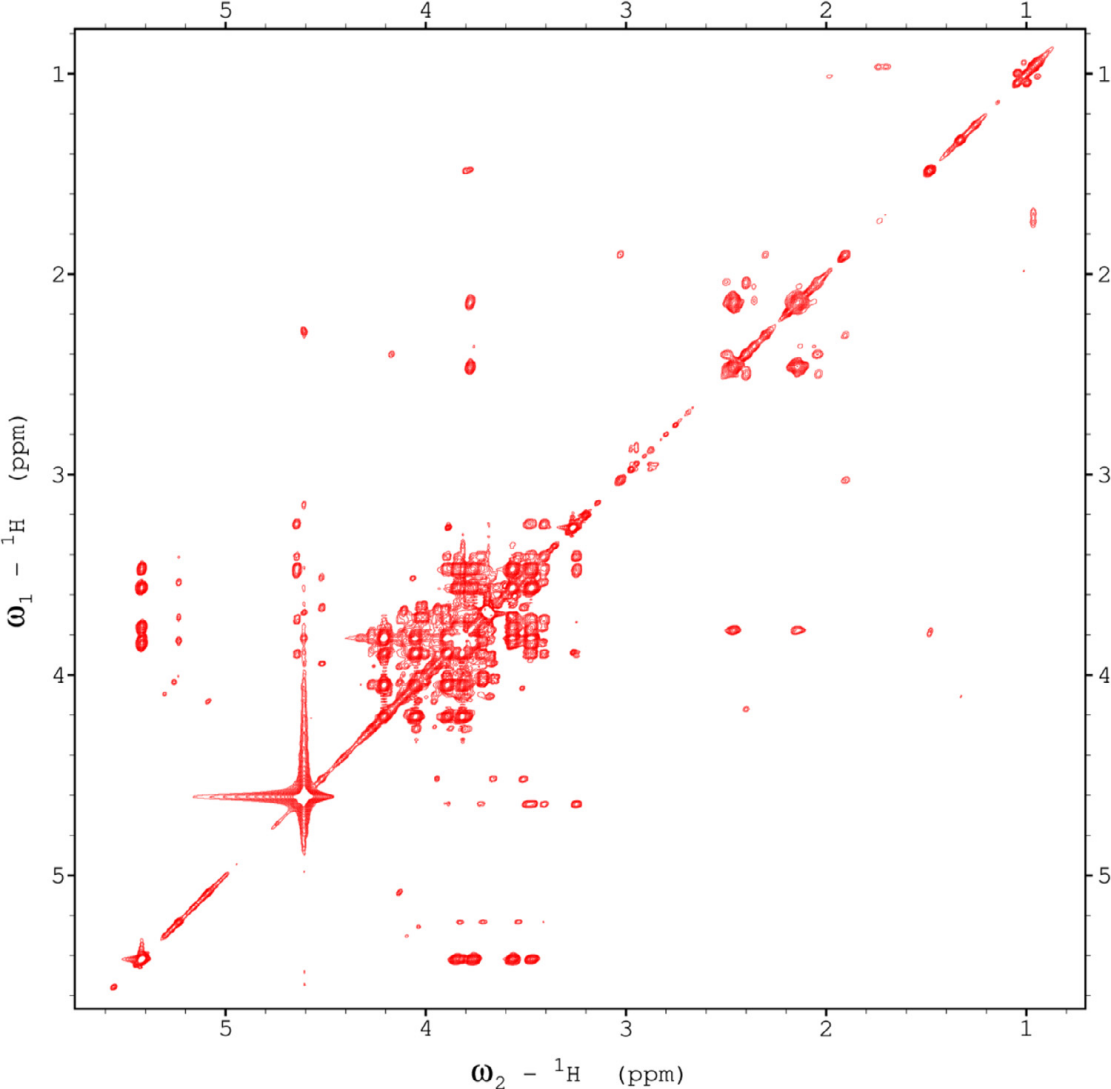
The 120ms zTOCSY NMR spectrum of the red beet water-soluble fraction.

Analysis of the 2D-NMR spectra for WSF at 40°C (Figs. 5-10) enabled the assignment of its resonances (in ppm) as follows, with ambiguous assignments given in paratheses and unassigned listed as “N.A.”:

α-Ara: C1/H1 110.3/5.085; C2/H2 N.A./4.132; C3/H3 N.A./3.958 ppm; C4-C5/H4-H5, N.A.
α-Glc(1): C1/H1 95.03/5.419; C2/H2 73.91/3.569; C3/H3 75.42/3.769; C4/H4 75.24/3.845; C5/H5 72.2/3.471, C6/H6_a,b_ 63.04/(3.788/3.849)
α-Glc(2): C1/H1 94.81/5.417; C2/H2 73.69/3.561; C3/H3 N.A./ 3.764; C4/H4 N.A./, C5/H5 72.19/3.816; C6/H6_a,b_ 65.92/N.A.
α-Glc(3) (sucrose): C1/H1 94.93/5.234; C2/H2 74.42/3.538; C3/H3 75.53/3.716; C4/H4 71.31/3.415; C5/H5 74.26/3.837; C6/H6_a,b_ N.A.
β-Fru*f* (sucrose): C1/H1_a,b_ N.A./(3.687/N.A.); C2, 106.5; C3/H3 N.A; C4/H4 79.46/3.954, C5-C6/H5-H6_a,b_ N.A.
β-Fru*f* (free): C1/H1_a,b_ 65.74/(3.568/3.589); C2, 104.3; C3/H3 77.43/N.A.; C4/H4 78.49/4.105; C5/H5 83.59/4.112; C6/H6_a,b_ N.A./(3.83/N.A.)
β-Fru*p*: C1/H1_a,b_ 66.89/(3.567/3.715); C2, 100.8; C3/H3 70.56/3.800; C4/H4 72.5/3.893; C5/H5 71.97/3.995; C6/H6_a,b_ 66.2/(3.702/4.027)
β-Glc(1): C1/H1 98.8/4.646; C2/H2 77.04/3.247; C3/H3 78.68/3.483; C4/H4 72.54/3.408; C5/H5 78.88/3.465; C6/H6_a,b_ 63.64/(3.722/3.894)
β-Glc(2): C1/H1 99.65/4.519; C2/H2 N.A./3.513; C3/H3N.A./3.666; C4/H4 71.4/3.943; C5/H5 75.17/3.909; C6/H6_a,b_ N.A./(3.262/N.A.)
β-X (unknown): C1/H1 94.99/4.818; C2-C5/H2-H5 N.A.; C6/H6_a,b_ 65.0/N.A.
Y (unknown): C1/H1 92.4/N.A.; C2/H2 79.31/4.209; C3/H3 77.25/4.052; C4/H4 84.2/3.891; C5/H5 65.21/3.818; C6/H6_a,b_ N.A.

**Fig. 11:**
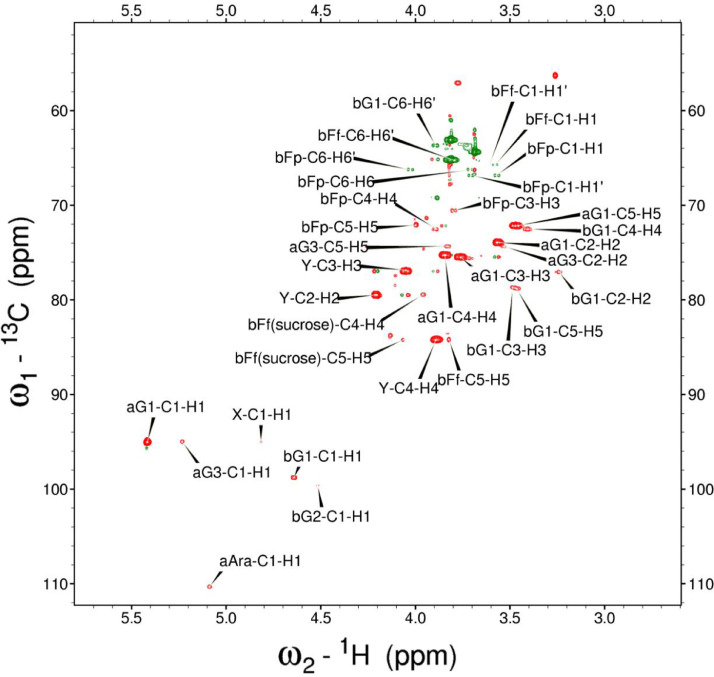
The multiplicity-edited HSQC NMR spectrum of the red beet pomace fraction with assignments. Red colored peaks indicate carbons with odd numbers of attached hydrogens, while green indicates a CH_2_ group.

**Fig. 12:**
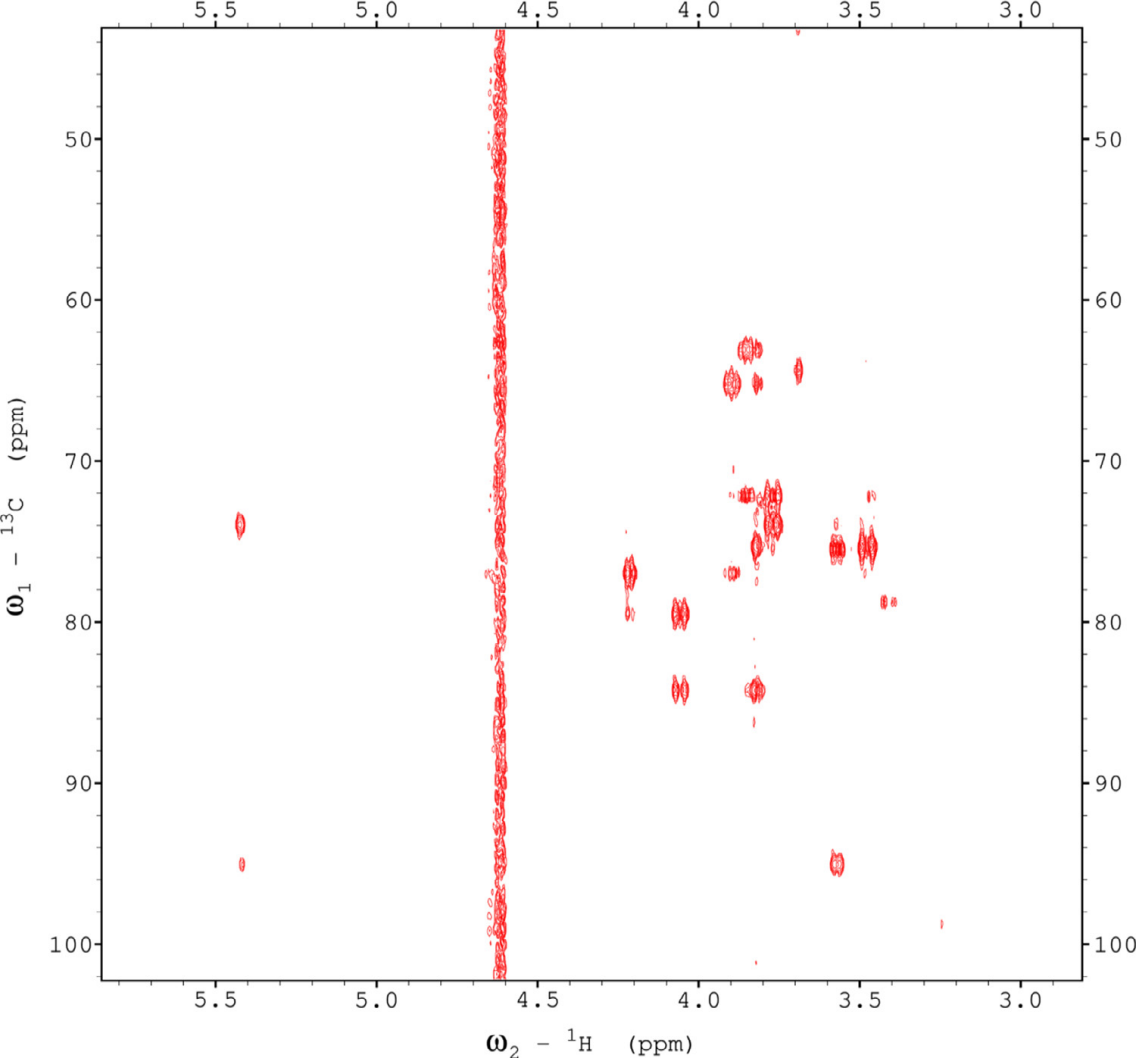
The H2BC NMR spectrum of the red beet pomace fraction.

**Fig. 13:**
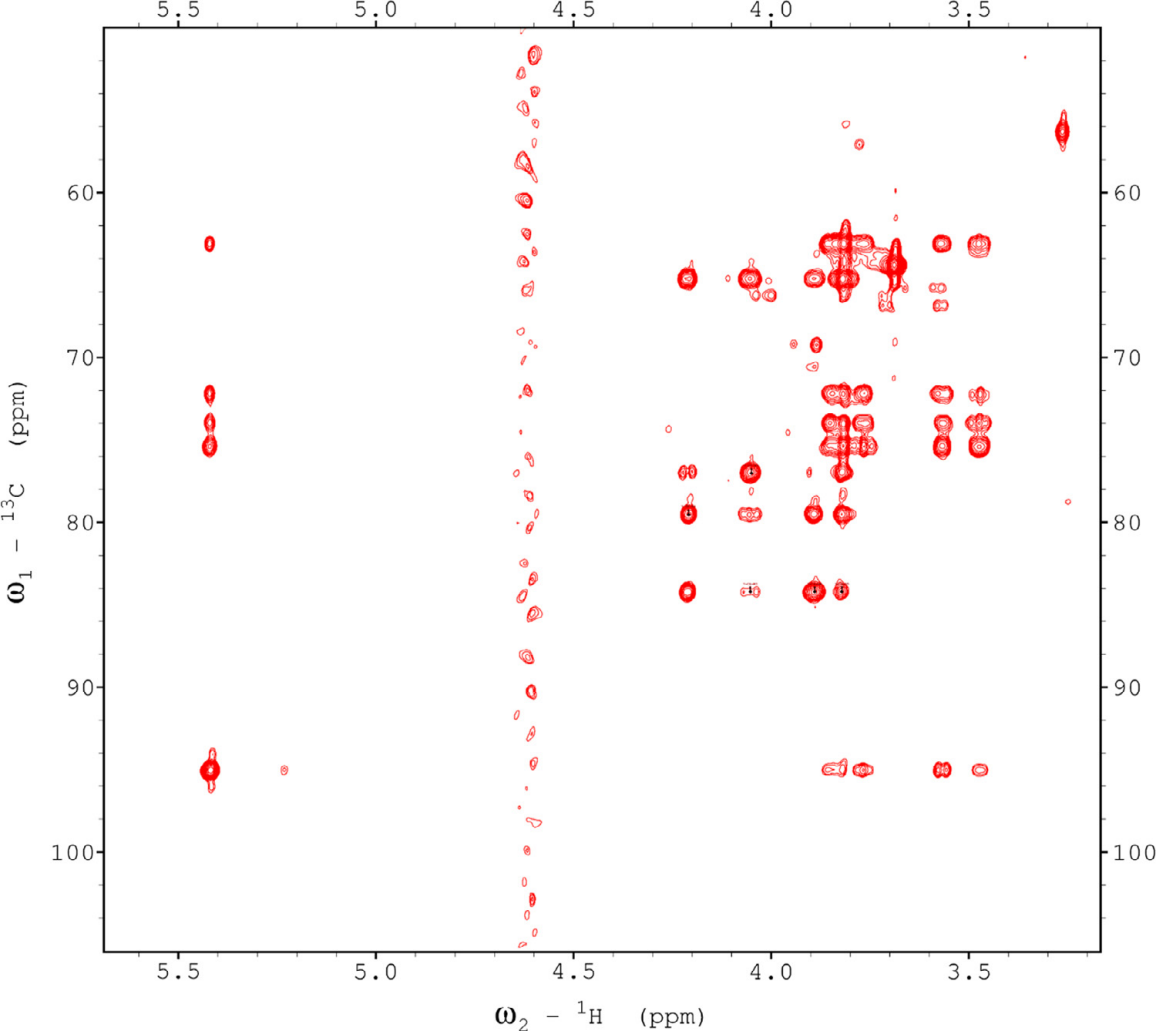
The 130ms HSQCTOCSY NMR spectrum of the red beet pulp fraction.

**Fig. 14:**
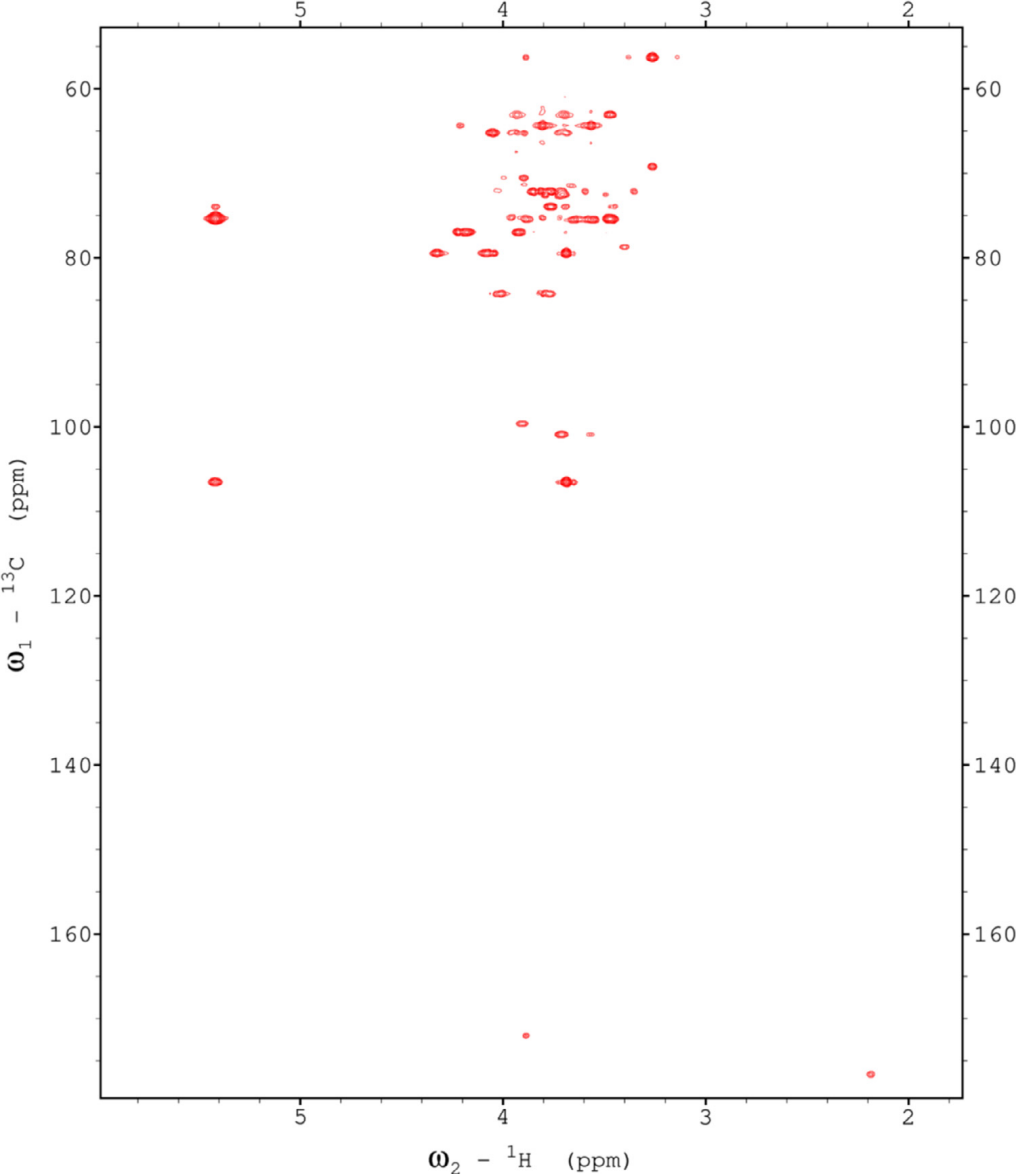
The 16 Hz HMBC NMR spectrum of the red beet pulp fraction.

**Fig. 15:**
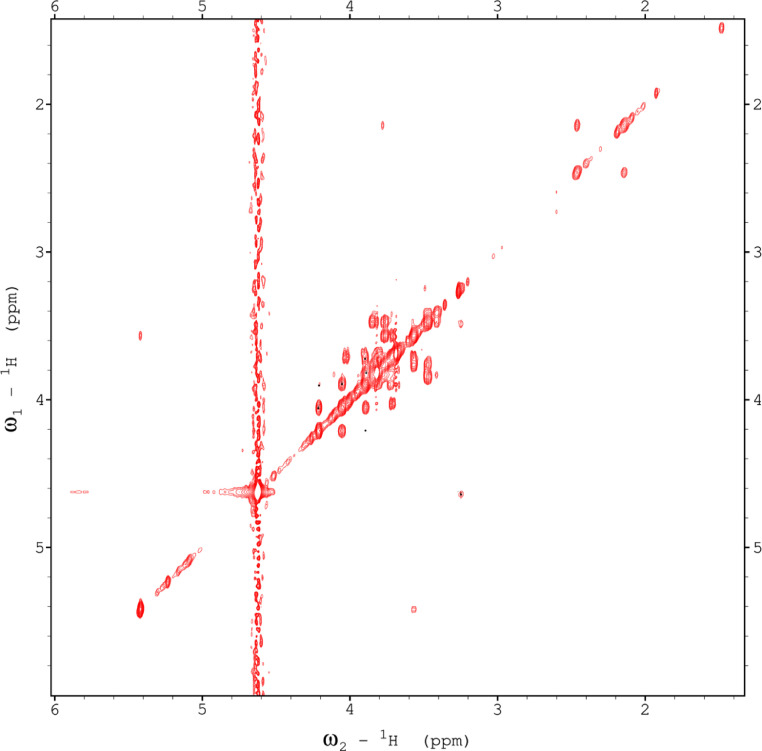
The 22 ms zTOCSY NMR spectrum of the red beet pulp fraction.

**Fig. 16:**
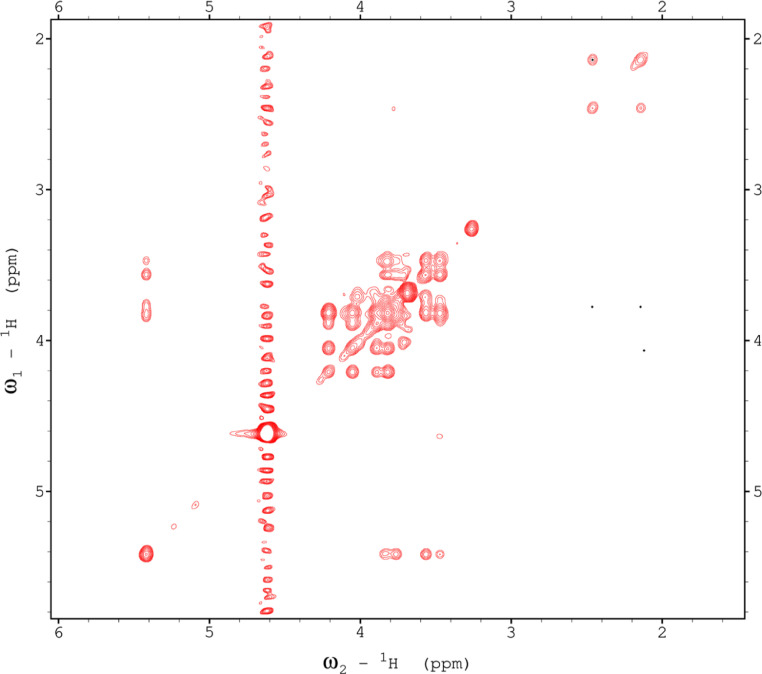
The 130ms zTOCSY NMR spectrum of the red beet pulp fraction.

Analysis of the 2D-NMR spectra for PF at 40°C (Figs. 11-16) enabled the assignment of its resonances (in ppm) as follows, with ambiguous assignments given in paratheses and unassigned listed as “N.A.”:

α-Ara: C1/H1 110.3/5.088; C2-C5/H2-H5 N.A.
α-Glc(1): 95.05/5.42; C2/H2 73.98/3.569; C3/H3 75.47/3.763; C4/H4 73.69/3.713; C5/H5 72.18/3.661; C6/H6 N.A.
α-Glc(3) (sucrose): C1/H1 94.99/5.232; C2/H2 74.37/3.53; C3-C4/H3-H4 N.A.; C5/H5 74.33/3.833; C6/H6 N.A.
β-Fru*f* (sucrose): C1/H1_a,b_ N.A.; C2/H2 106.6/N.A.; C3/H3 79.44/N.A.; C4/H4 84.2/3.961; C5/H5 N.A./4.062; C6/H6_a,b_ N.A./(3.687/N.A.)
β-Fru*f* (free): C1/H1_a,b_ 65.73/(3.58/3.608); C2-C5/H2-H5 N.A.; C6/H6_a,b_, 65.27/(3.795/N.A.)
β-Fru*p*: C1/H1_a,b_ 66.83/(3.57/3.709); C2/H2 100.9/N.A.; C3/H3 70.6/3.803; C4/H4 72.46/3.893; C5/H5 72.12/4.00; C6/H6_a,b_ 66.26/(3.712/4.026)
β-Glc(1): C1/H1 98.8/4.642; C2/H2 77.05/3.246; C3/H3 78.7/3.486; C4/H4 72.51/3.404; C5/H5 78.87/3.464; C6/H6_a,b_ 63.68/(3.893/N.A.)
β-Glc(2): C1/H1 99.65/4.516; C2/H2 N.A./3.51; C3-C4/H3-H4 N.A.; C5/H5 N.A./3.904; C6/H6 N.A.
β-X (unknown): C1/H1 95.03/4.816; C2-C6/H2-H6 N.A.
Y (unknown): C1/H1 N.A.; C2/H2 79.52/4.211; C3/H3 76.98/4.054; C4/H4 84.24/3.892; C5/H5 N.A./3.82; C6/H6 N.A.

**Fig. 17:**
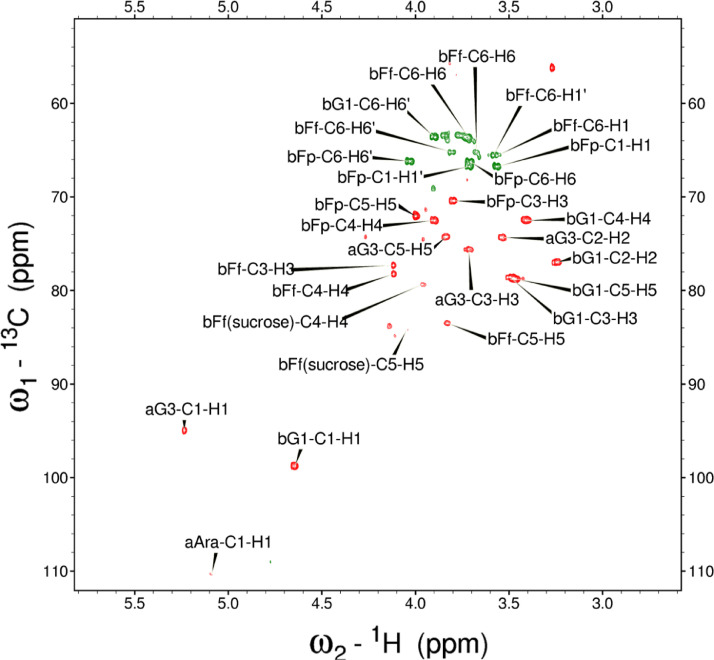
The multiplicity-edited HSQC NMR spectrum of the red beet water-insoluble fraction with assignments. Red colored peaks indicate carbons with odd numbers of attached hydrogens, while green indicates a CH_2_ group.

**Fig. 18:**
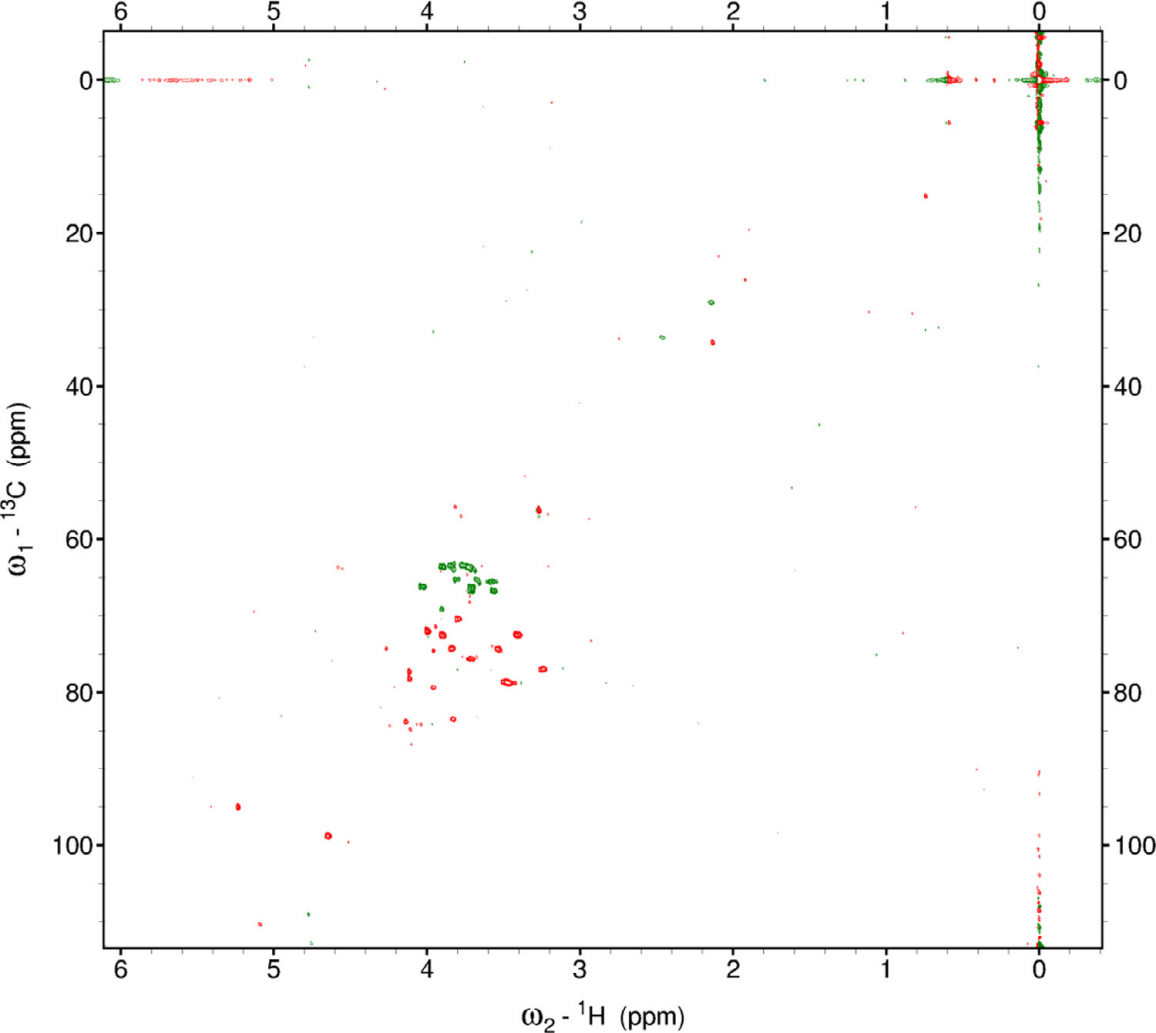
The full multiplicity-edited HSQC spectrum of the red beet water-insoluble fraction. Red colored peaks indicate carbons with odd numbers of attached hydrogens, while green indicates a CH_2_ group This HSQC spectrum reveals the presence of small aliphatic peaks in the upfield region which are similar to those in the other fractions.

**Fig. 19:**
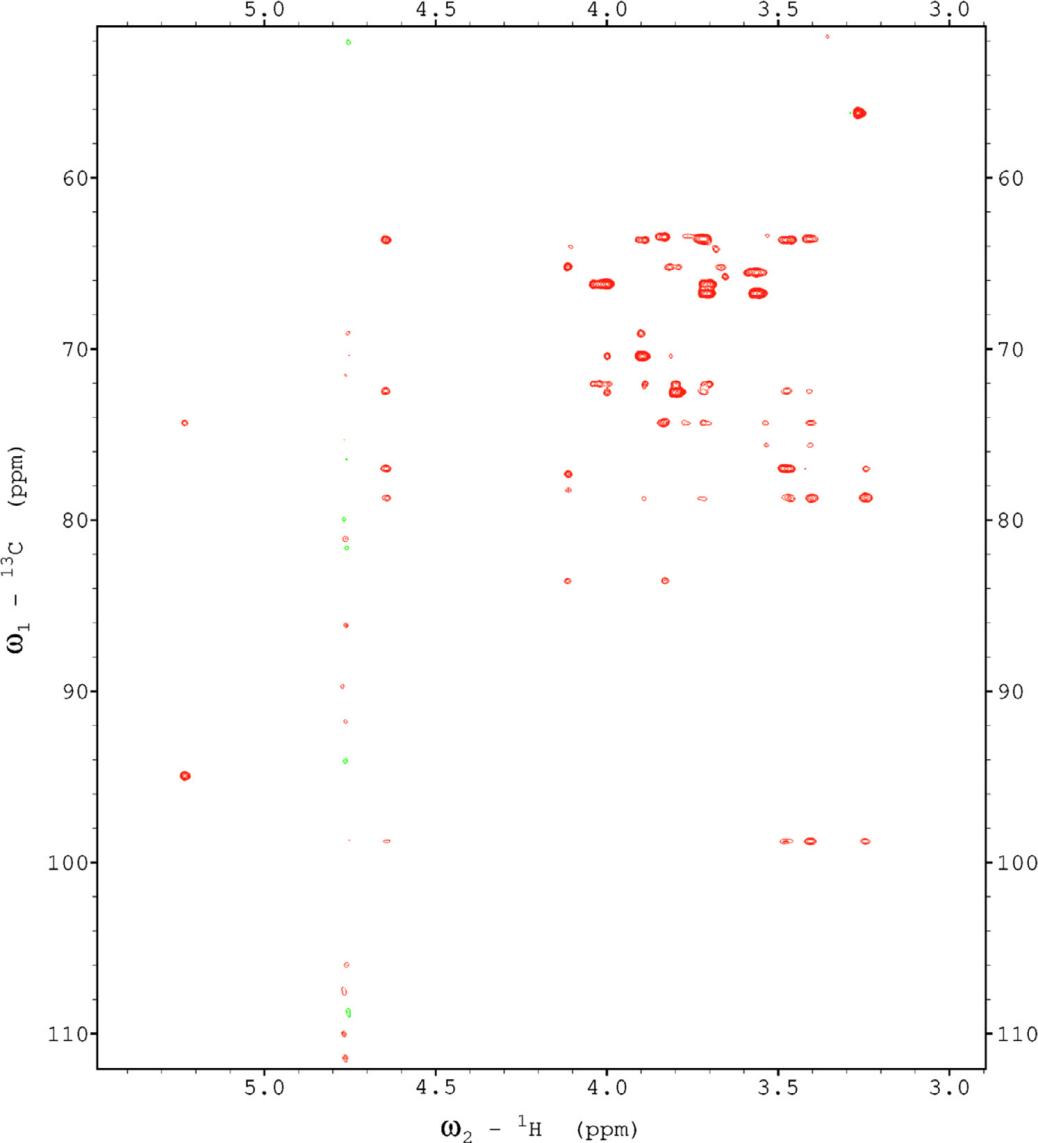
The 130 ms HSQCTOCSY NMR spectrum of the red beet water-insoluble fraction.

**Fig. 20:**
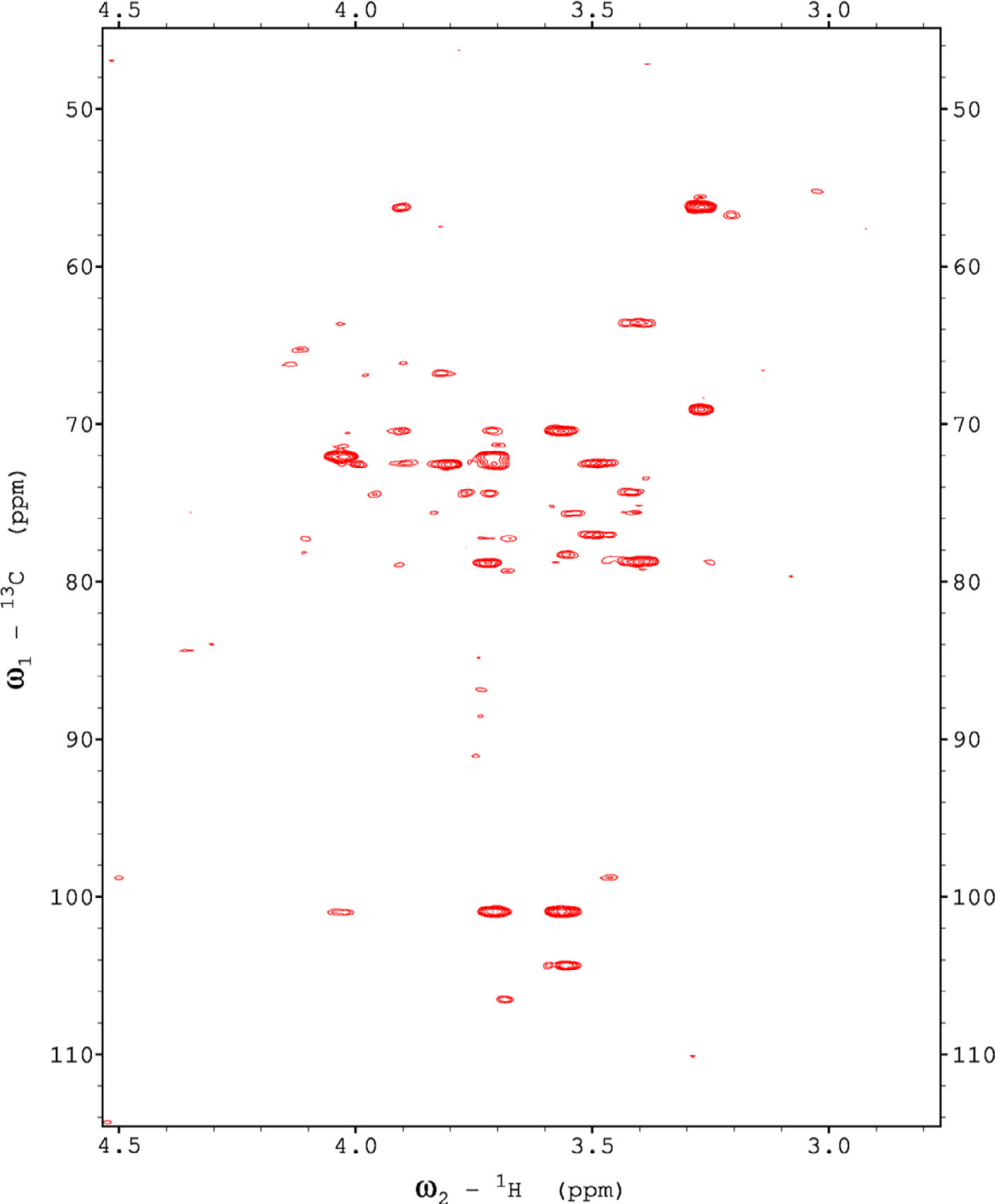
The 5 Hz HMBC NMR spectrum of the red beet water-insoluble fraction.

**Fig. 21:**
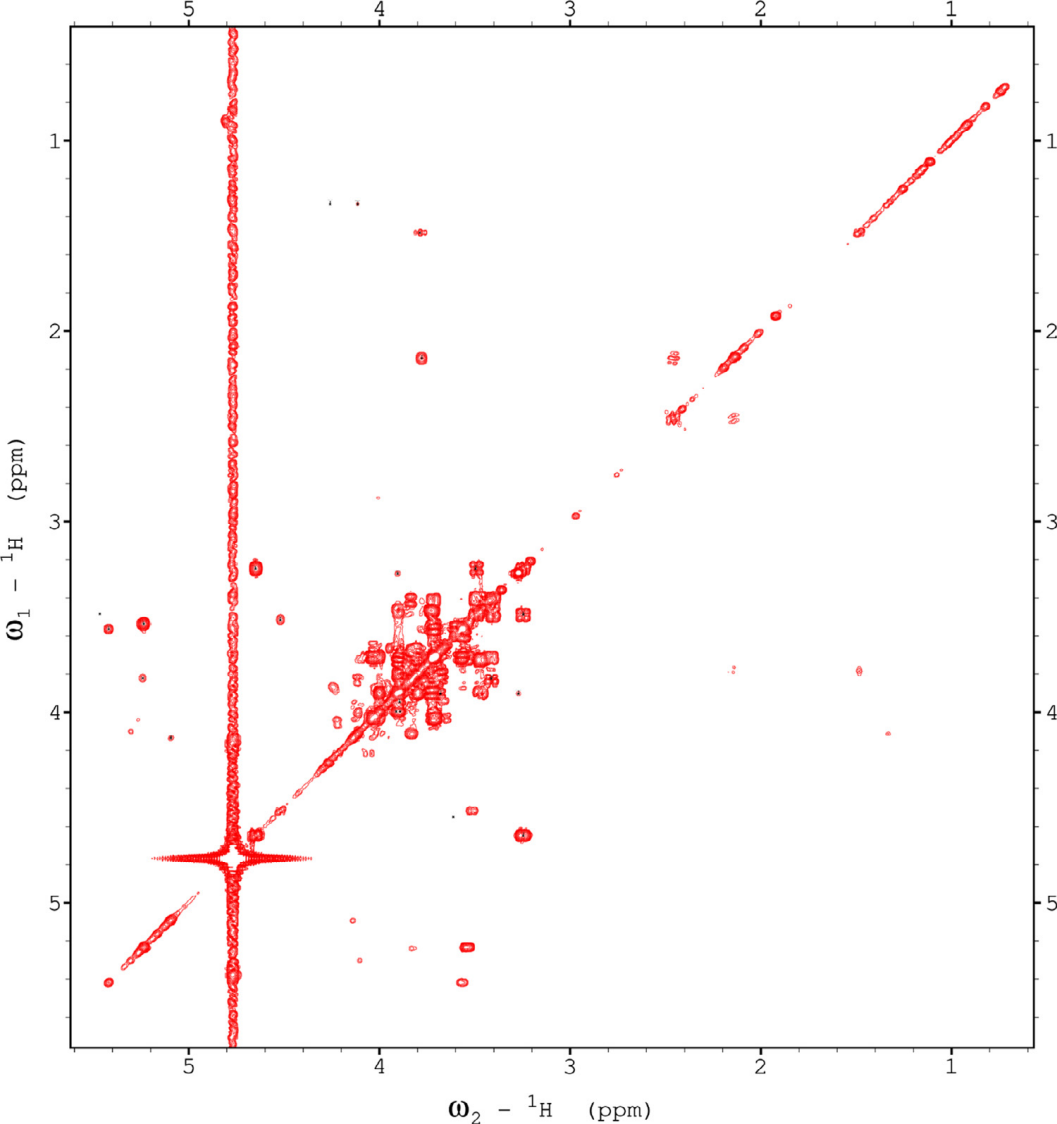
The gCOSY NMR spectrum of the red beet water-insoluble fraction.

**Fig. 22:**
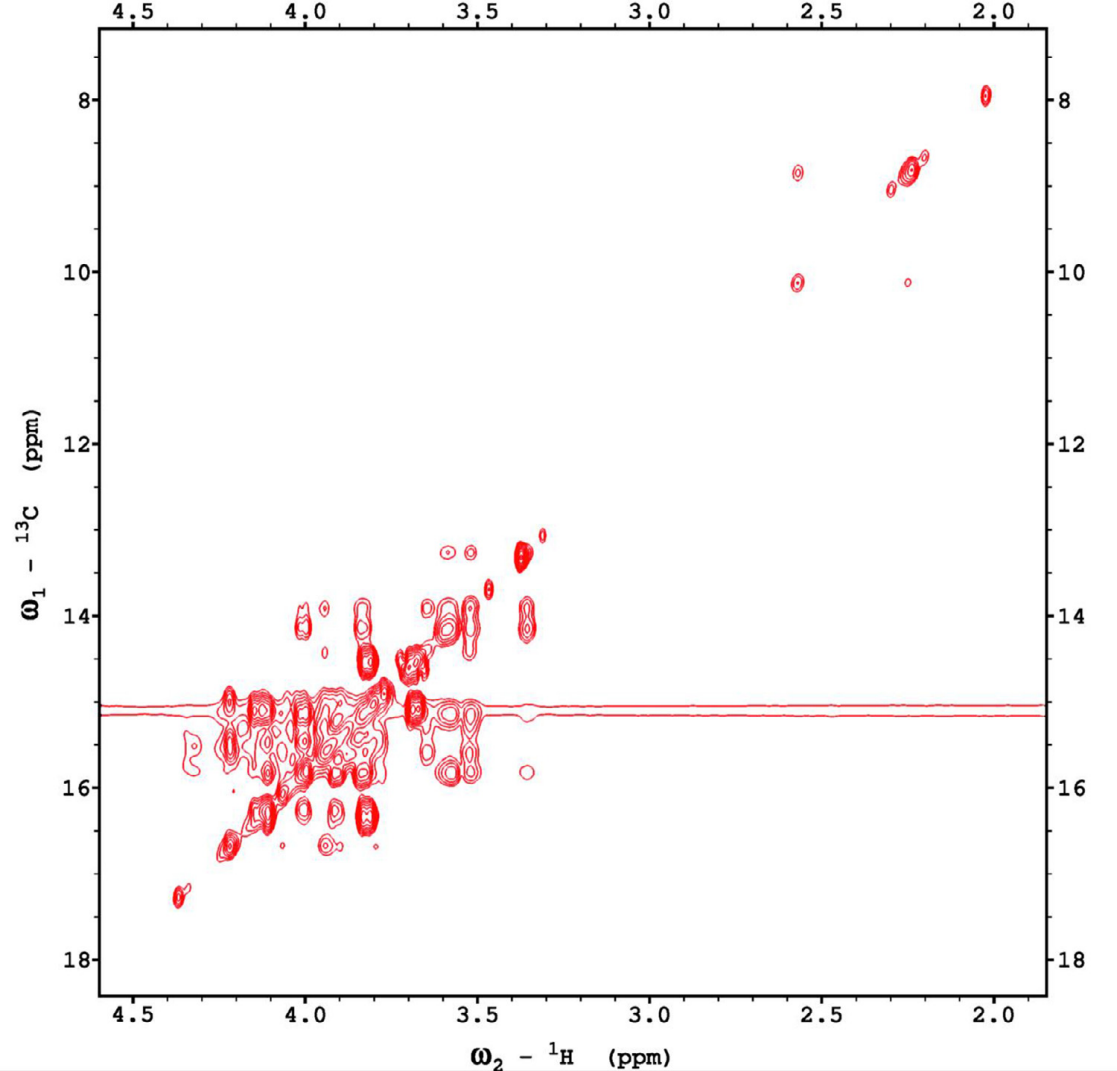
The 130 ms zTOCSY NMR spectrum of the red beet water-insoluble fraction.

Analysis of the 2D-NMR spectra for WIF at 40°C (Figs. 17-22) enabled the assignment of its resonances (in ppm) as follows, with ambiguous assignments given in paratheses and unassigned listed as “N.A.”:

α-Ara: C1/H1 110.3/5.091; C2/H2 N.A./4.136, C3-C5/H3-H5 N.A.
α-Glc(2): C1/H1 N.A./5.42; C2/H2 N.A./3.564; C3-C6/H3-H6 N.A.
α-Glc(3) (sucrose): C1/H1 94.92/5.235; C2/H2 74.34/3.536; C3/H3 75.65/3.714; C4/H4 72.53/3.409; C5/H5 74.31/3.834; C6/H6_a,b_ 63.41/(3.771/N.A.)
β-Fru*f* (sucrose): C1/H1_a,b_ 65.79/(3.668/N.A.); C2/H2 106.5/N.A.; C3/H3 N.A./4.101; C4/H4 79.37/3.965; C5/H5 N.A.; C6/H6_a,b_ 64.15/(3.677/N.A.)
β-Fru*f* (free): C1/H1_a,b_ 65.56/(3.557/3.591); C2/H2 104.4/N.A.; C3/H3 77.35/4.115; C4/H4 78.27/4.114; C5/H5 83.52/3.829; C6/H6_a,b_ 65.11/(3.684, 3.808)
β-Fru*p*: C1/H1_a,b_ 66.74/(3.566/3.713); C2/H2 100.9/N.A.; C3/H3 70.39/3.801; C4/H4 72.54/3.896; C5/H5 72.06/3.999; C6/H6_a,b_ 66.22/(3.707/4.028).
β-Glc(1): C1/H1 98.76/4.644; C2/H2 77.0/3.244; C3/H3 78.7/3.484; C4/H4 72.48/3.408; C5/H5 77.24/3.465; C6/H6_a,b_ 63.6/(3.725/3.898)
β-Glc(2): C1/H1 N.A./4.519; C2/H2 N.A./3.514; C3/H3 N.A.; C4/H4 N.A./3.947; C5/H5 N.A./3.901; C6/H6_a,b_ N.A./(3.271/N.A.)

**Fig. 23:**
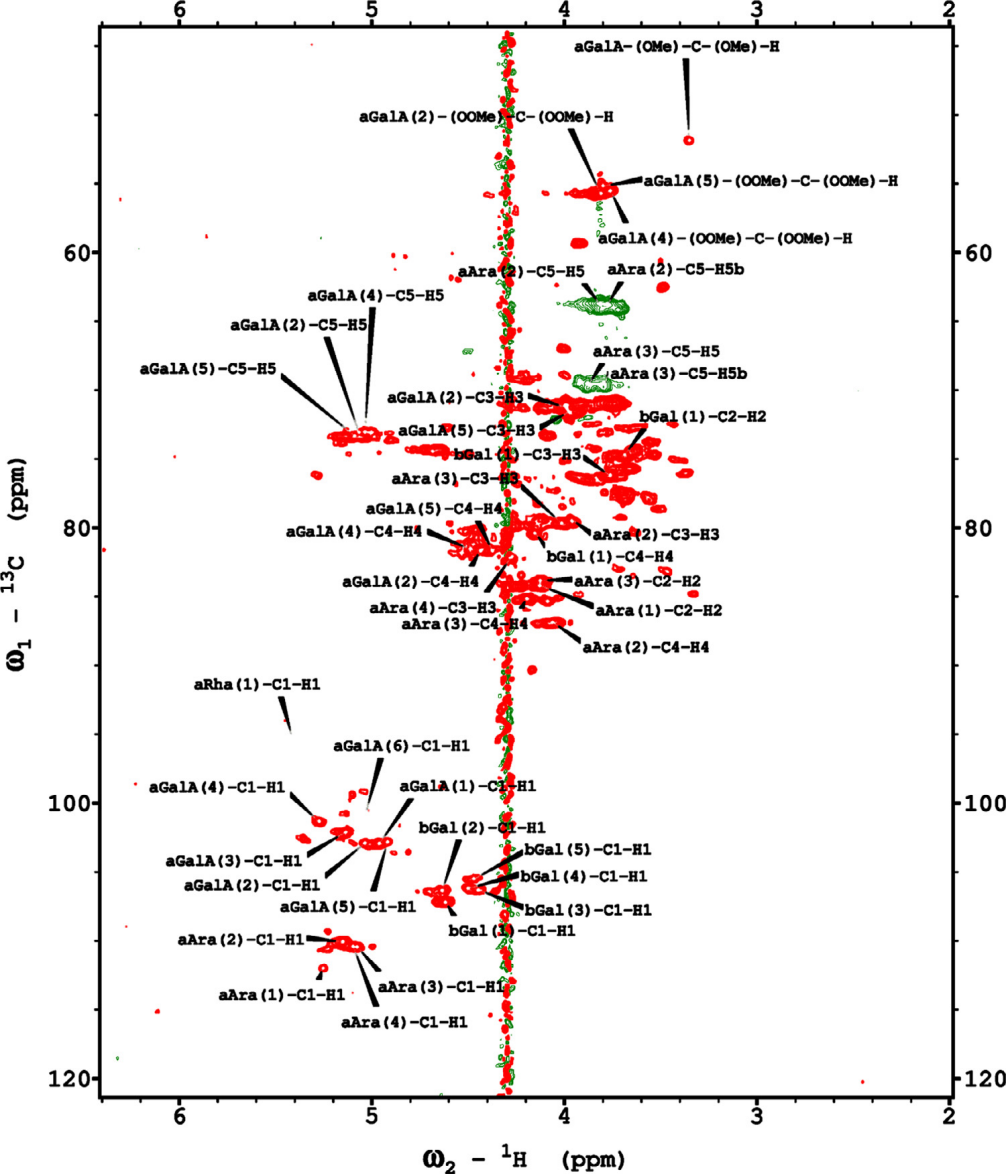
The multiplicity-edited, sensitivity enhanced HSQC spectrum (75°C) of the sugar resonance region (excluding methyl region) of the red beet pectin. Red colored peaks indicate carbons with odd numbers of attached hydrogens, while green indicates a CH_2_ group.

**Fig. 24:**
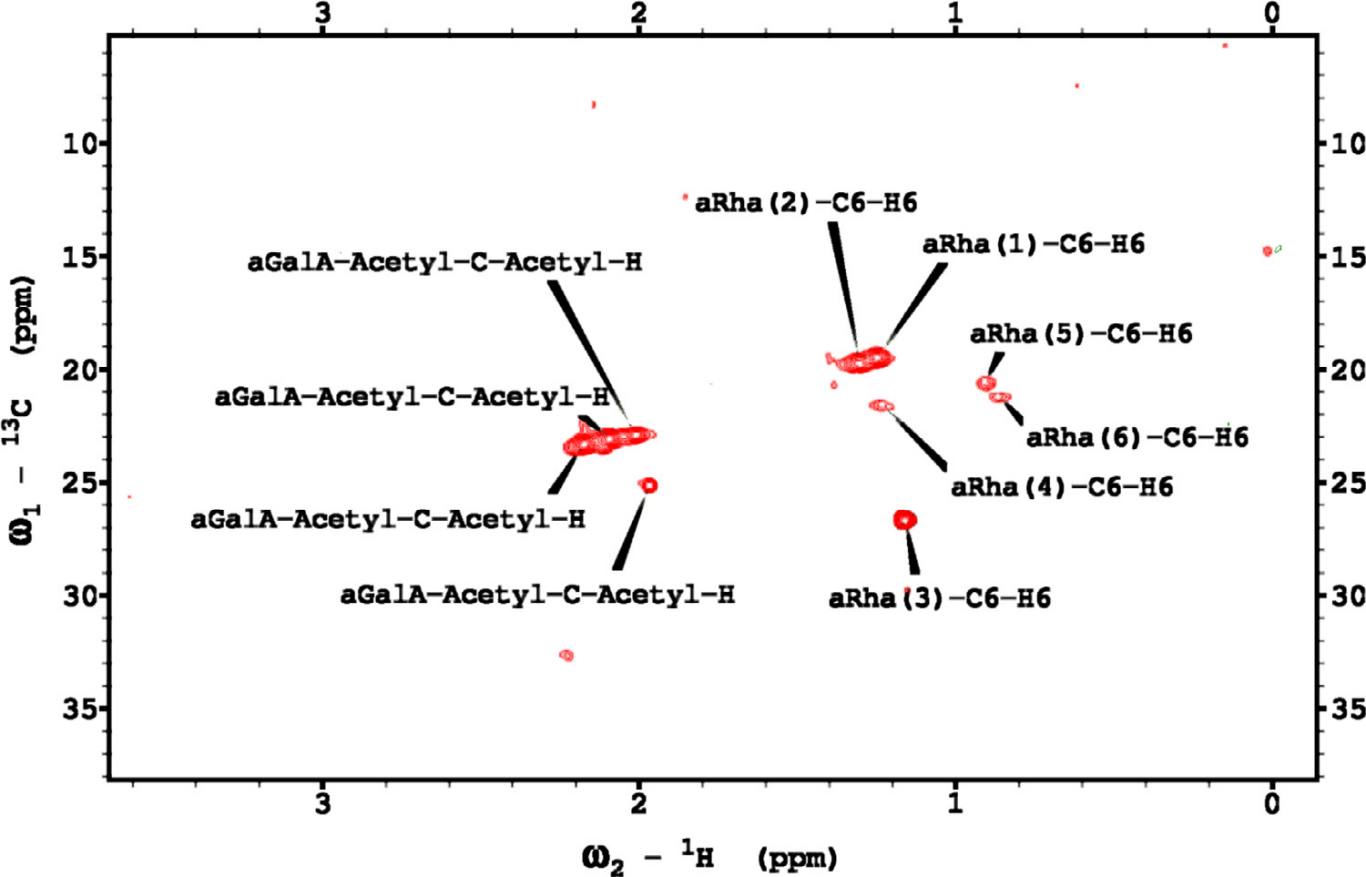
The multiplicity-edited, sensitivity enhanced HSQC spectrum (75°C) of methyl region of the red beet pectin covering the rhamnose C6H and the acetyl groups.

**Fig. 25:**
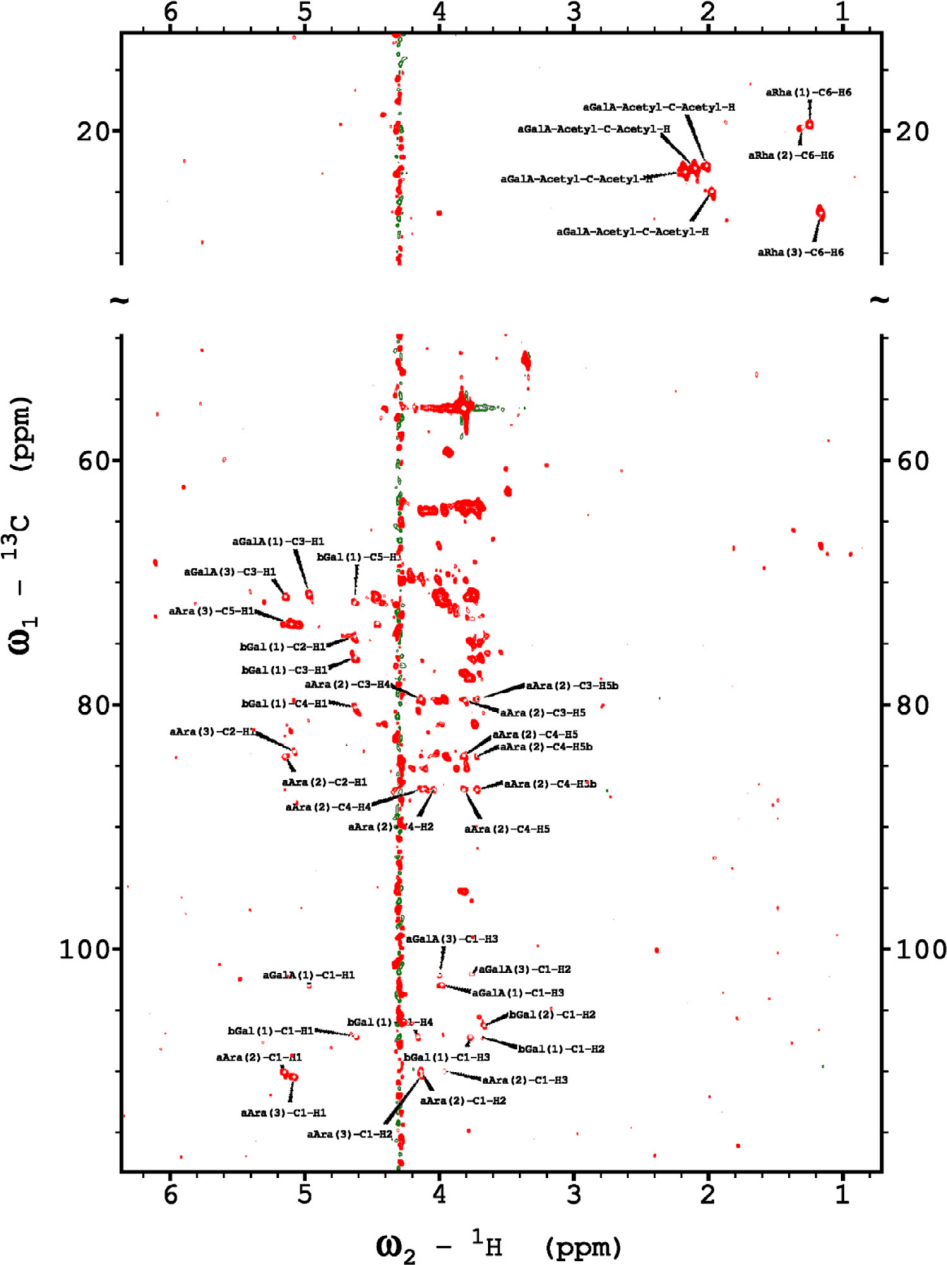
The full 150 ms HSQCTOCSY spectrum (75°C) of the red beet pectin.

**Fig. 26:**
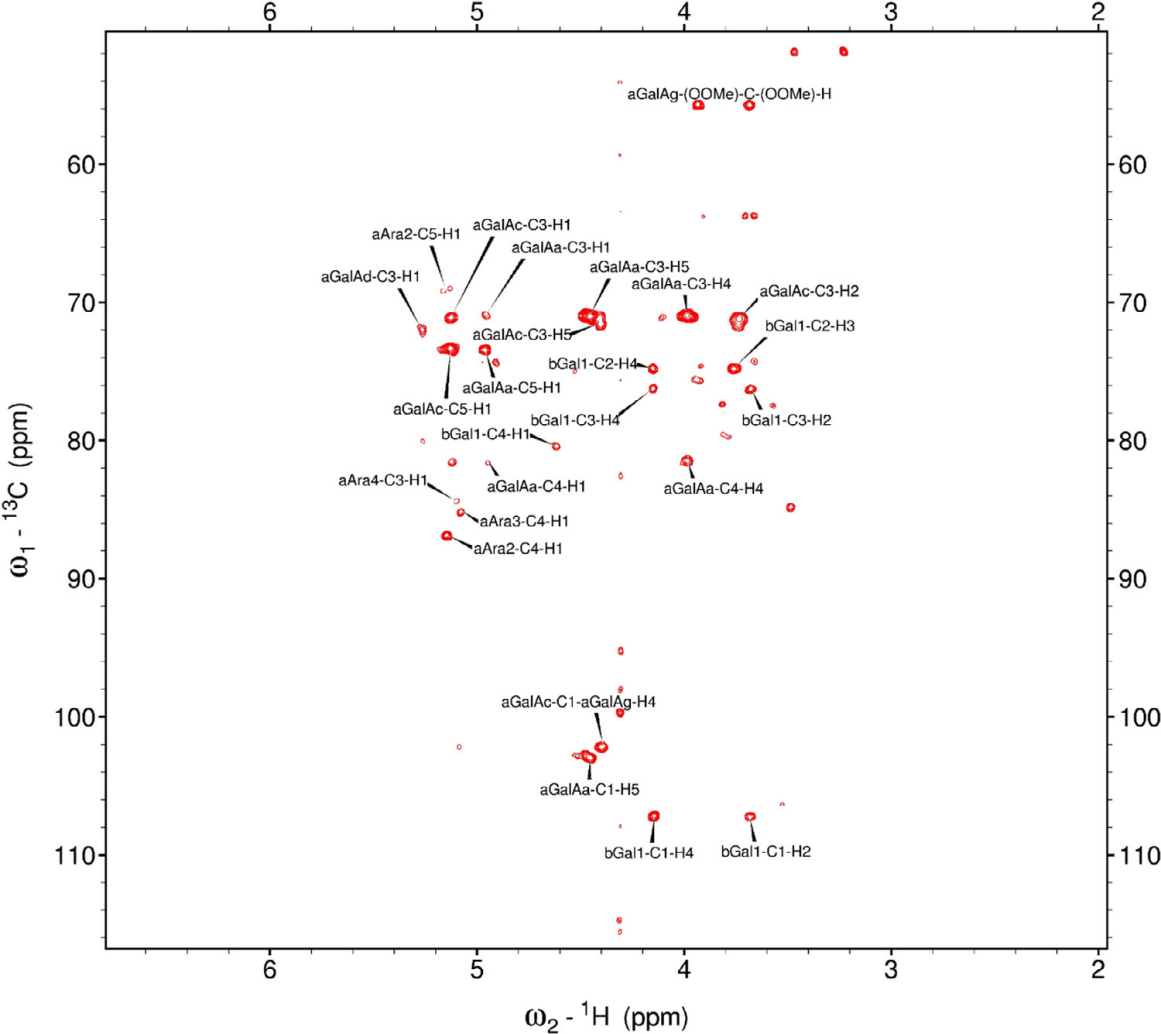
The 16 Hz gHMBC spectrum (75°C) of the anomeric and sugar ring resonances of the red beet pectin.

**Fig. 27:**
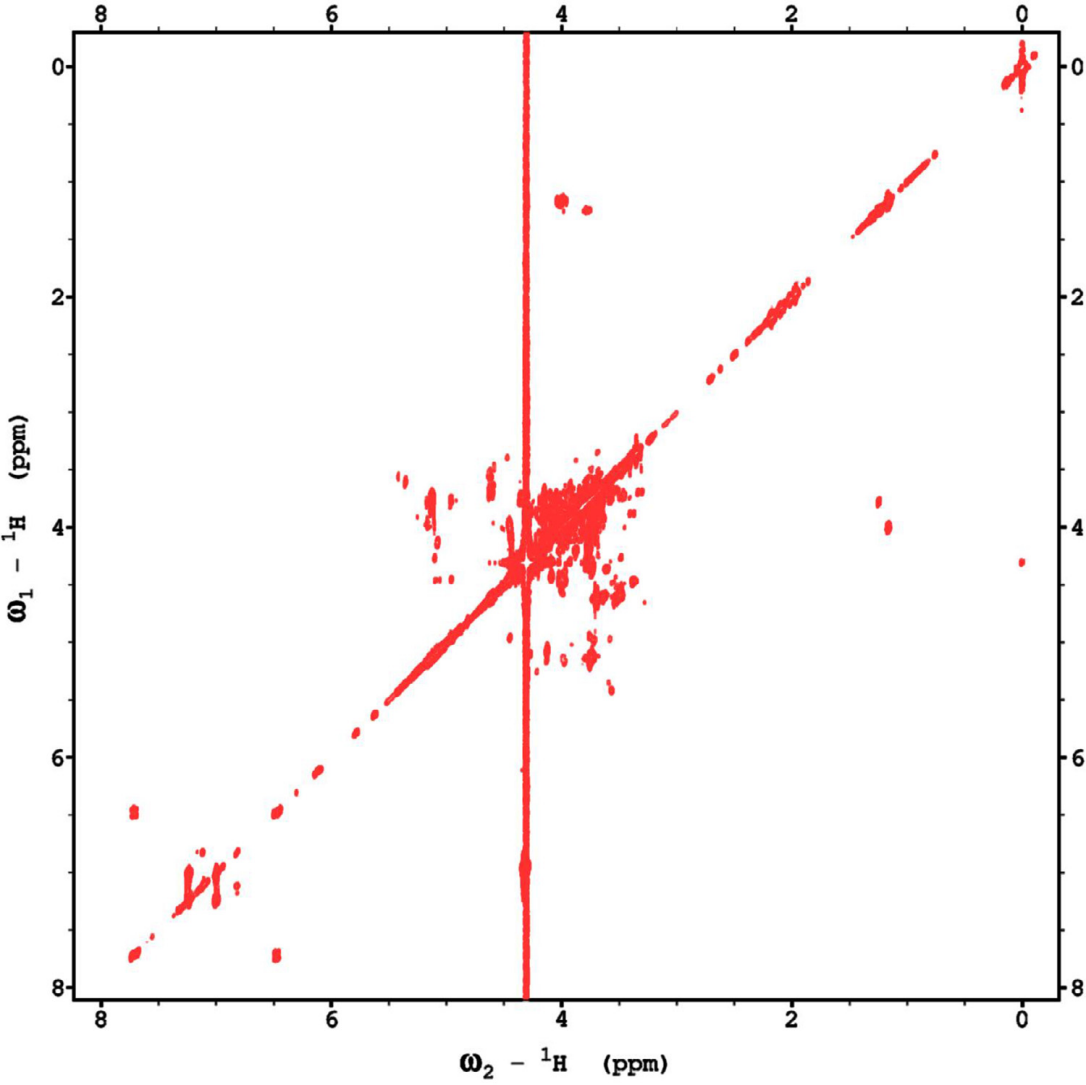
The full gCOSY spectrum (75°C) of the red beet pectin sample.

**Fig. 28:**
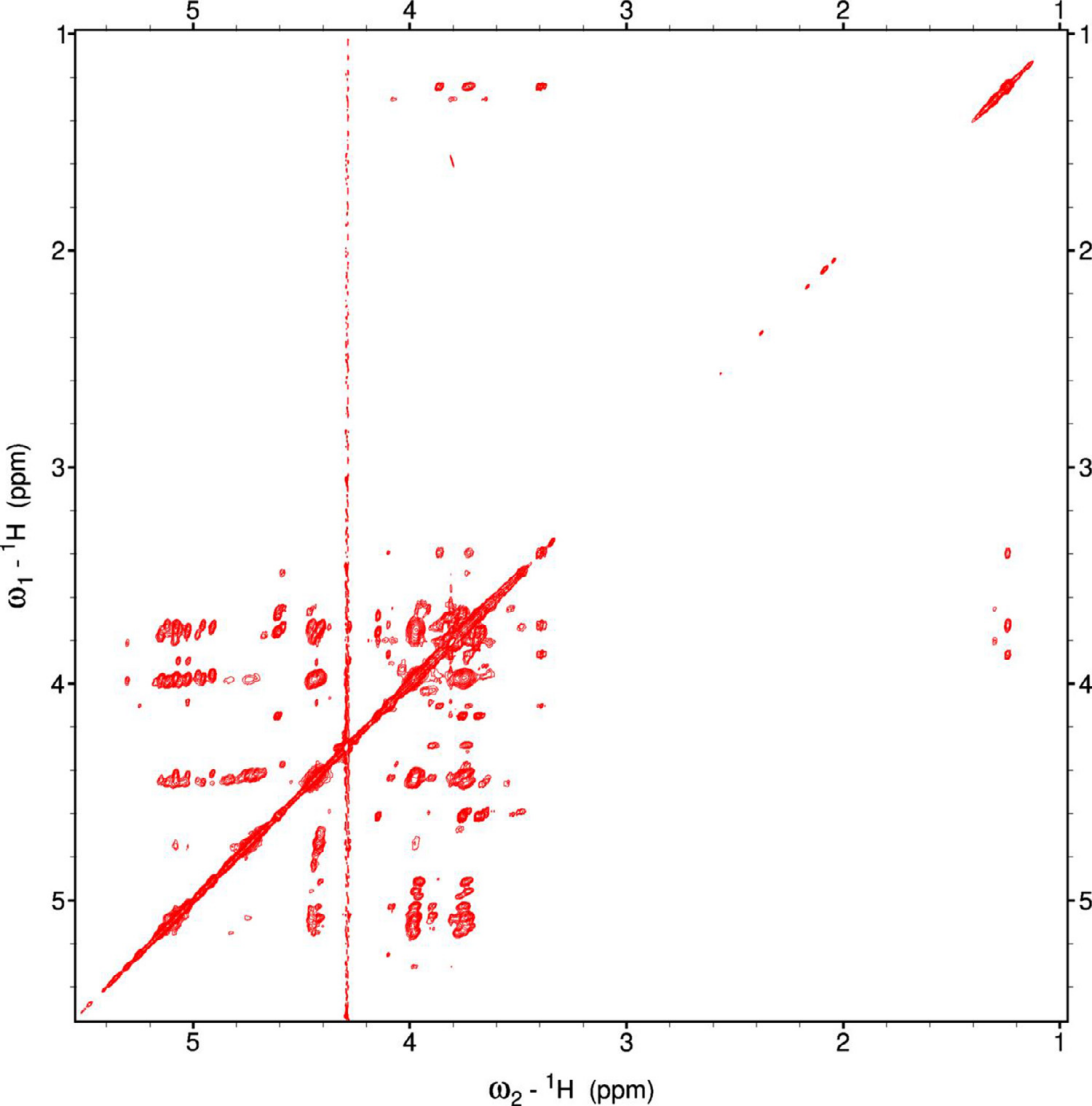
The 150 ms zTOCSY spectrum (75°C) of the red beet pectin sample.

NOTES: (a) In all of the following images, peak labels are defined as follows:

Analysis of the 2D-NMR spectra for red beet pectin at 75°C (Figs. 23-28) enabled the assignment of its resonances (in ppm) and anomeric couplings (in Hz), with ambiguous assignments given in paratheses and unassigned listed as “N.A.” and tentative (*):

α-GalA (1): C1/H1 102.9/4.60 (^1^*J*_CH_=173 Hz); C2/H2 N.A./3.738; C3/H3 70.93/3.979; C4/H4 81.4/4.078; C5/H5 73.37/4.455; C6 N.A.
α-GalA (2): C1/H1 102.9/5.036 (^1^*J*_CH_=175 Hz); C2/H2 N.A./3.734; C3/H3 71.22/3.984; C4/H4 81.66/4.449; C5/H5 73.07/5.083; C6 173.6; Methyl Ester 55.75/3.817
α-GalA (3): C1/H1 102.1/5.130 (^1^*J*_CH_=172 Hz); C2/H2 N.A./3.744; C3/H3 71.24/3.991; C4/H4 N.A./4.183; C5/H5 73.31/4.405; C6: N.A.
α-GalA (4): C1/H1 101.3/5.267 (^1^*J*_CH_=171 Hz); C2/H2 N.A./4.433; C3/H3 72.09/3.993; C4/H4 81.51/4.487; C5/H5 73.07/5.037; C6 173.3; Methyl Ester 55.52/3.762
α-GalA (5): C1/H1 102.8/4.907 (^1^*J*_CH_=170 Hz); C2/H2 N.A./3.739, C3/H3 71.6/3.968; C4/H4 81.66/4.407; C5/H5 73.32/5.101; C6 172.8; Methyl Ester 55.41/3.807
α-GalA (6): C1/H1 100.6/5.029 (^1^*J*_CH_=170 Hz); C2-C6/H2-H6 N.A.
α-GalA (7)*: C1/H1 99.49/5.096 (^1^*J*_CH_=169 Hz); C2-C6/H2-H6 N.A.
α-Ara (1): C1/H1 112/5.261; C2/H2 84.3/4.105, C3/H3 N.A./4.018; C4/H4 N.A./4.297; C5/H5_a,b_ N.A.
α-Ara (2): C1/H1 110/5.154 (^1^*J*_CH_=174 Hz); C2/H2 84.16/4.081; C3/H3 79.49/3.964 C4/H4 86.52/4.114; C5/H5_a,b_ 64.46/(3.827, 3.728)
α-Ara (3): C1/H1 110.3/5.084 (^1^*J*_CH_=175 Hz); C2/H2 83.78/4.136; C3/H3 79.55/4.256; C4/H4 85.14/4.194; C5/H5_a,b_ 70.82/(3.885, 3.810)
α-Ara (4): C1/H1 110.4/5.109 (^1^*J*_CH_=173 Hz); C2/H2 N.A./4.107; C3/H4 82.83/4.294; C4-C5/H4-H5 N.A.
-4)-β-Gal (1): C1/H1 107.2/4.620 (^1^*J*_CH_=159 Hz); C2/H2 74.71/3.684; C3/H3 76.21/3.764; C4/H4 80.31/4.152; C5/H5 71.64/3.935; C6/H6_a,b_ N.A./(3.532, N.A.)
t-β-Gal (2): C1/H1 106.3/4.616 (^1^*J*_CH_=164 Hz); C2/H2 N.A./3.666; C3/H3 N.A.; C4/H4 N.A./3.932; C5-C6/H5-H6 N.A.
β-Gal (3): C1/H1 106.3/4.457 (^1^*J*_CH_=157 Hz); C2/H2 N.A./3.984; C3/H3 N.A./3.734; C4-C6/H4-H6 N.A.
β-Gal (4): C1/H1 105.5/4.470 (^1^*J*_CH_=57 Hz); C2-C6/H2-H6 N.A.
β-Gal (5): C1/H1 106.2/4.50 (^1^*J*_CH_=N.D.); C2-C6/H2-H6 N.A.
β-GlcA*: C1/H1 98.88/4.634 (^1^*J*_CH_=159 Hz); C2-C6/H2-H5 N.A.
α-Rha (1): C1/H1 95.14/5.414 (^1^*J*_CH_=172 Hz); C2/H2 N.A./4.104; C3/H3 N.A./3.88; C4/H4 74.97/3.386; C5/H5 71.83/3.778; C6/H6 19.49/1.248,
α-Rha (2): C1/H1 N.A.; C2/H2 N.A./4.094; C3/H3 N.A.; C4/H4 N.A./3.64; C5/H5 65.33/3.852; C6/H6 19.72/1.303
α-Rha (3): C1-C4/H1-H4 N.A.; C5/H5 67.06/4.00; C6/H6 26.68/1.163
α-Rha (4): C1-C4/H1-H4 N.A.; C5/H5 N.A./3.768; C6/H6 21.56/1.234
α-Rha (5): C1-C5/H1-H5 N.A.; C6/H6 20.53/0.908
α-Rha (6): C1-C5/H1-H5 N.A.; C6/H6 21.19/0.866

aG = α-Glucose, bG = β-Glucose, aFf = α-Fructofuranose, bFf = β-Fructofuranose, bFp = β-Fructopuranose, while X and Y refer to unassigned sugar resonances.
(b) The parenthetical numerals are for identification purposes only, and do not refer to locations in the polysaccharides.

## 2 Experimental Design, Materials and Methods

### 2.1 Sample preparation

Red beet samples (pomace, water-soluble, water-insoluble and pectin) were prepared according to Hotchkiss et al. [1].

NMR Methods.

The four polysaccharide fractions derived from red beet, PF, WSF, WIF and pectin, were dissolved in D_2_O with d_4_-trimethylsilylpropanoic acid (TMSP) added as an internal reference standard and sodium azide (NaN_3_) added as a preservative. Their NMR spectra were acquired at 40°C and 75°C. The 1D-^1^H NMR spectra were acquired with a relaxation delay of 1-2 s, a 45° pulse angle, and spectral widths of 6.5-10 ppm, and 32k data points. The 1D-^13^C spectra were acquired semi-quantitatively using proton decoupling only during the acquisition after a 45° pulse, an acquisition time of 0.87 s and a relaxation delay of 1 s. The spectral width was 250 ppm. The T1 relaxation times were not measured. The gradient-enhanced COSY and zTOCSY experiments were acquired with spectral widths of 5.8, 6.5 or 10 ppm in both dimensions, using 4k points in the directly-detected dimension, and 400 indirectly-detected increments. The zTOCSY experiments were acquired with mixing times of 22, 120, and130 ms. HSQC, HSQC-TOCSY, HMBC and H2BC heteronuclear experiments had spectral widths of either 6.5 or 10 ppm in the directly-detected (^1^H) dimension, while the indirectly-detected dimensions varied as follows: the HSQC and HSQC-TOCSY experiments covered 130 ppm, the HMBC experiments covered 240 ppm, and the H2BC experiments, 80-120 ppm. The signal averaging ranged from 64-128 transients per increment, with the exception of the HMBC and H2BC experiments which were averaged over 256-512 transients per increment. The indirect dimensions collected over 300-400 increments for most experiments, but the constant time H2BC used 108-178 increments. The HSQC spectra were multiplicity-edited to distinguish between carbons with odd or even numbers of attached hydrogens. The HSQC-TOCSY experiments had mixing times of 80-130 ms. HMBC experiments were acquired using C-H coupling constants of 5, 8, 10 and 16 Hz. All NMR spectra were processed using [2] and visualized using UCSF Sparky [3].

## Ethics Statements

Mention of trade names or commercial products in this publication is solely for the purpose of providing specific information and does not imply recommendation or endorsement by the U.S. Department of Agriculture. USDA is an equal opportunity provider and employer.

## CRediT authorship contribution statement

**Gary D. Strahan:** Methodology, Investigation, Writing – original draft. **Arland T. Hotchkiss:** Conceptualization, Methodology, Investigation, Writing – original draft, Supervision, Funding acquisition. **Senghane Dieng:** Investigation, Resources. **Julie Hirsch:** Conceptualization, Supervision, Funding acquisition.

## Declaration of Competing Interest

The authors declare that they have no known competing financial interests or personal relationships that could have appeared to influence the work reported in this paper.

## Data Availability

Strahan-Hotchkiss-RedBeet-PF (Original data) (Food Data Central) Strahan-Hotchkiss-RedBeet-WSF (Original data) (Food Data Central) Strahan-Hotchkiss-RedBeet-Pectin (Original data) (Food Data Central) Strahan-Hotchkiss-RedBeet-WIF (Original data) (Food Data Central)
